# Computational Pathology Reveals Extracellular-Matrix Imaging Biomarkers of Therapy Response in Preclinical Breast Cancer

**DOI:** 10.3390/cancers18162603

**Published:** 2026-08-12

**Authors:** Stelios Lamprou, Styliana Georgiou, Triantafyllos Stylianopoulos, Chrysovalantis Voutouri

**Affiliations:** 1 AnaBioSi-Data Ltd., Athalassas Ave. 176, MyOffice 204, Strovolos 2025, Cyprus; stelios@anabiosi-data.com (S.L.); georgiou.styliana.1@ucy.ac.cy (S.G.); 2 Cancer Biophysics Laboratory, Department of Mechanical Engineering, University of Cyprus, P.O. Box 20537, Nicosia 1678, Cyprus; tstylian@ucy.ac.cy

**Keywords:** breast cancer, extracellular matrix, collagen, hyaluronic acid, immunofluorescence, computational pathology, deep learning, therapy response

## Abstract

**Background/Objectives:** Histological biomarkers of therapy response remain incompletely defined in preclinical breast cancer models. We evaluated whether quantitative immunofluorescence imaging and multimodal machine learning (ML) could identify image-derived tissue biomarkers associated with response in a 4T1 murine breast cancer therapy-response setting. **Methods:** We analysed 1696 immunofluorescence images across nine immunofluorescence staining panels: aPDL1, alpha-smooth muscle actin-CD31, alpha-smooth muscle actin-Ki67, CD3-CD31, CD8-Ki67, collagen–hyaluronic acid (HA), granzyme B-CD8, HMGB1, and pimonidazole/hypoxia. A Python image-analysis algorithm extracted 117 RGB-channel, intensity, morphometric, and cross-channel spatial features per image. The modelling framework included image-only deep learning (DL), tabular ML on extracted histological features, and image-plus-tabular gated fusion. Models were evaluated using stratified cross-validation, train-fold-only class balancing, bootstrap confidence intervals, permutation testing, nested feature-selection sensitivity analysis, and grouped leakage controls. **Results:** Histological staining panels classified treatment-response status across the nine-panel benchmark. Collagen–HA was the strongest staining (AUC 0.954), followed by alpha-smooth muscle actin-Ki67 (AUC 0.851) and hypoxia (AUC 0.837). DL achieved the best performance in eight of nine stainings. In 346 collagen–HA images, combined collagen and HA features achieved AUC 0.848, collagen-only features AUC 0.833, and hyaluronic-acid-only features AUC 0.805. In 104 animals with matched hypoxia and collagen–hyaluronic-acid images, extracellular-matrix features achieved AUC 0.985, hypoxia-only features achieved AUC 0.929, and adding hypoxia did not improve extracellular-matrix-only prediction. **Conclusions:** Quantitative extracellular-matrix imaging provides a strong preclinical signal for therapy-response stratification in 4T1 breast cancer. The findings are hypothesis-generating and require independent preclinical and human validation before clinical translation.

## Simple Summary

Breast cancer treatment response is influenced not only by cancer cells but also by the surrounding tissue environment. In this preclinical study, we tested whether information contained in immunofluorescence images from a 4T1 mouse breast cancer model could help distinguish therapy responders from non-responders. We analysed 1696 images covering nine tissue-marker panels and compared image-based deep learning with machine learning models trained on quantitative image features. The strongest signal came from extracellular-matrix markers, especially collagen and hyaluronic acid. In animals with matched hypoxia and extracellular-matrix images, collagen and hyaluronic-acid features outperformed hypoxia, and adding hypoxia did not improve prediction. These findings suggest that quantitative tissue architecture is a useful preclinical biomarker source, but the results require validation in independent animal experiments and human tumour tissue before clinical use.

## 1. Introduction

Breast cancer is a major global health burden and remains the most commonly diagnosed cancer and the leading cause of cancer death among women worldwide. In 2022, approximately 2.3 million new cases and 670,000 deaths were attributed to female breast cancer, accounting for 11.6% of all cancer diagnoses and 6.9% of cancer deaths globally [1]. Despite improvements in screening, molecular classification, and systemic therapy, treatment response remains heterogeneous, particularly in aggressive and metastatic disease. This variability reflects not only malignant-cell-intrinsic features but also the tumour microenvironment (TME), which is increasingly recognised as an active regulator of tumour progression and therapeutic response [2,3].

The breast cancer TME comprises immune cells, cancer-associated fibroblasts, endothelial and perivascular cells, adipocytes, soluble mediators, and the extracellular matrix (ECM). Bidirectional signalling between these compartments can promote tumour-cell survival, invasion, angiogenesis, immune evasion, and treatment resistance. Mechanistically, cytokine and growth-factor signalling can establish immunosuppressive and pro-angiogenic states, whereas ECM deposition, crosslinking, and alignment increase tissue stiffness and activate integrin-dependent focal-adhesion signalling. Experimental breast cancer models have shown that collagen crosslinking can enhance integrin signalling and malignant progression, while dense collagen can restrict T-cell proliferation and cytotoxic activity, thereby linking matrix architecture to both tumour mechanics and antitumour immunity [4,5].

Therapy response in breast cancer is therefore shaped by the combined effects of cancer-cell states and the surrounding tissue environment. Immune-checkpoint blockade and other systemic therapeutic strategies have changed oncology practice, but many patients do not derive durable benefit, and preclinical models remain essential for identifying mechanisms of response and resistance [6,7,8]. The 4T1 murine breast tumour model is useful in this setting because it is tumorigenic, immunologically active, metastatic, and widely used for breast cancer immunology and therapeutic testing [9,10]. Current biomarker approaches are based largely on genomic analyses and remain incomplete. Among histological markers, programmed death ligand 1 immunohistochemistry, tumour mutational burden, and microsatellite instability can stratify response in selected settings, but their predictive value varies across tumour types, assays, and therapeutic contexts [11,12,13,14]. Tissue biomarkers measurable from standard histology or immunohistochemistry could therefore provide a practical bridge between mechanistic biology and translational treatment stratification.

Apart from cancer-cell-intrinsic states, the ECM is a plausible source of predictive information. Collagen deposition, fibre organisation, matrix stiffening, and HA accumulation can alter tumour mechanics, interstitial pressure, vascular perfusion, drug penetration, and immune-cell localisation [15,16,17,18,19,20,21,22,23,24]. Prior work from this research group and others has helped define how collagen, HA, solid stress, and vascular compression create physical barriers to perfusion and therapy delivery, including in breast cancer and other solid tumour models [19,20,21,22,23,24]. These studies provide a mechanistic rationale for treating matrix architecture as a quantifiable biomarker rather than only as a histological background feature [25]. Hypoxia provides a related axis: low oxygen tension can promote immune suppression, alter metabolism, induce programmed death ligand 1, and contribute to treatment resistance [26,27,28]. Dense ECM can also compress vessels and reduce perfusion, thereby linking matrix remodelling to hypoxic tissue states [19,20,21,22,23,24].

Digital pathology offers a route to the reproducible and scalable quantification of these TME features. Classical ML can use interpretable handcrafted features, such as intensity, object morphology, and spatial overlap, whereas DL can learn higher-order image patterns directly from histology [29,30,31,32,33,34,35,36,37]. However, most computational pathology studies focus on diagnostic classification, mutation prediction, or prognosis from haematoxylin-and-eosin whole-slide images rather than multi-staining immunohistochemistry in preclinical therapy-response experiments [29,30,31,32,33,34,38]. Here, we developed a multimodal computational pathology framework for preclinical oncology immunohistochemistry. The framework separated image-only DL from tabular ML trained on Python-extracted histological features and from image-plus-tabular DL fusion, then applied this architecture to nine staining panels, ECM channel decomposition, and paired-animal-level ECM/hypoxia fusion.

## 2. Materials and Methods

### 2.1. Study Design and Dataset

**Figure 1:**
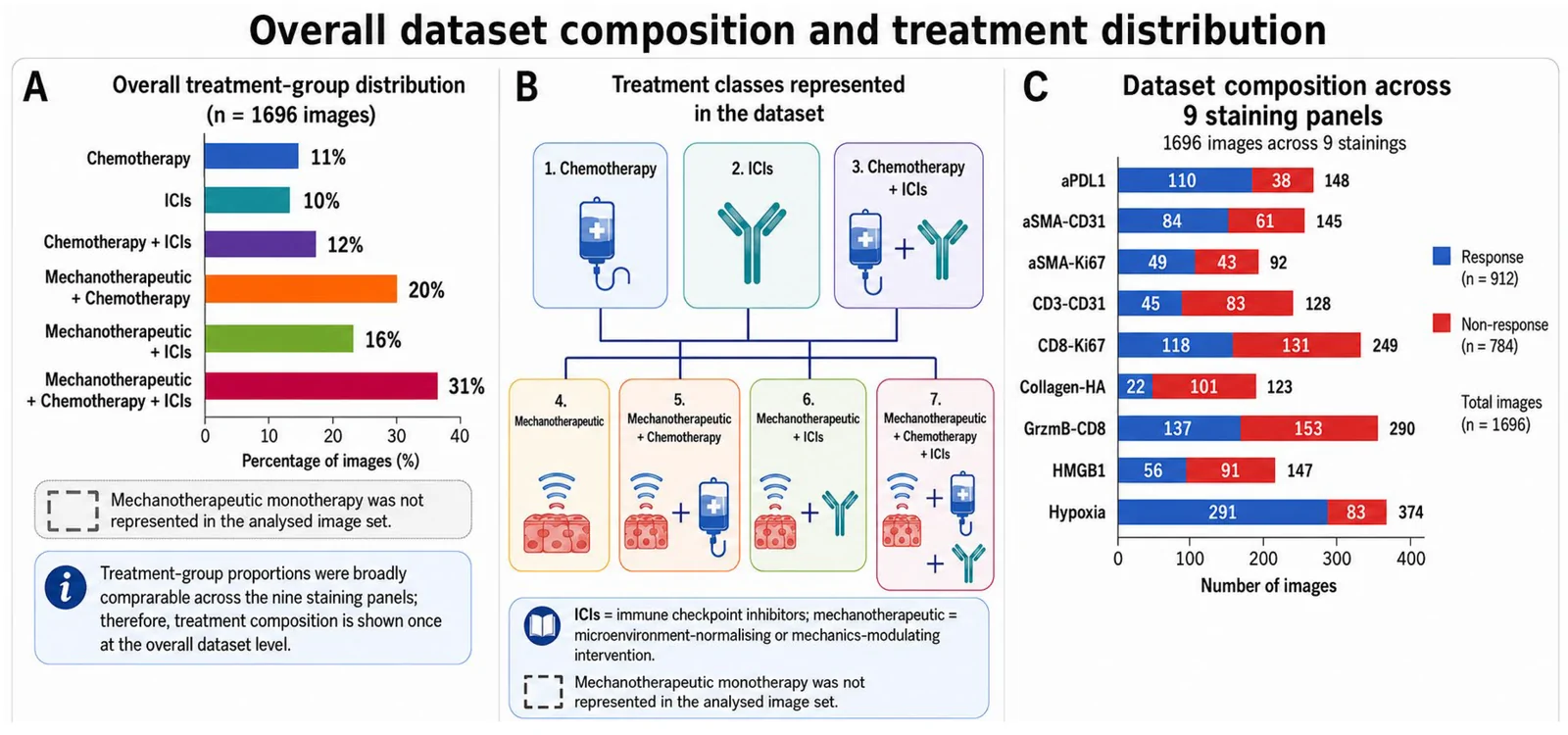
Overall dataset composition and treatment distribution. (**A**) Overall treatment-group distribution in the analysed preclinical 4T1 fluorescence immunohistochemistry image dataset (n = 1696). Treatment composition is shown once at the overall dataset level because proportions were broadly comparable across the nine staining panels. The represented treatment groups were chemotherapy (11%), immune-checkpoint inhibitors (ICIs; 10%), chemotherapy plus ICIs (12%), mechanotherapeutic plus chemotherapy (20%), mechanotherapeutic plus ICIs (16%), and mechanotherapeutic plus chemotherapy plus ICIs (31%). Mechanotherapeutic monotherapy was not represented in the analysed image set. (**B**) Schematic overview of the treatment classes represented in the dataset, including single-agent, dual-combination, and triple-combination treatment strategies. (**C**) Dataset composition across the nine staining panels, showing Response and Non-Response image counts and the overall total of 1696 images. The complete figure was generated using GPT-Image 2.0 to merge panels; Panel A (Python + GPT-Image 2.0), Panel B (GPT-Image 2.0), and Panel C (Python v.3.14.7).

This retrospective computational pathology study used fluorescence immunohistochemistry images from a preclinical 4T1 murine breast cancer therapy-response experiment. The endpoint was binary classification of therapy Response vs. Non-Response, as defined in the source experimental datasets as shown in Figure 1. The source experiment included untreated or control animals and animals allocated to chemotherapy, immune-checkpoint inhibition, chemotherapy plus immune-checkpoint inhibition, mechanotherapeutic combinations with chemotherapy and/or immune-checkpoint inhibition, and triple-combination treatment. Mechanotherapeutics were drugs repurposed to remodel the TME, reduce excessive ECM deposition, alleviate tumour stiffness and solid stress, and improve perfusion and hypoxia. In the analysed image set, treatment-group proportions were broadly comparable across the nine staining panels; therefore, treatment composition is summarised once at the overall dataset level rather than repeated separately for each staining. The overall treatment distribution in the analysed image set was chemotherapy 11%, immune-checkpoint inhibition 10%, chemotherapy plus immune-checkpoint inhibition 12%, mechanotherapeutic plus chemotherapy 20%, mechanotherapeutic plus immune-checkpoint inhibition 16%, and mechanotherapeutic plus chemotherapy plus immune-checkpoint inhibition 31%; mechanotherapeutic monotherapy was not represented. The full benchmark dataset comprised 1696 images across nine staining panels: anti-PD-L1 (aPDL1) (n = 148; 110 Response, 38 Non-Response), alpha-smooth muscle actin (aSMA)-CD31 (n = 145; 84 Response, 61 Non-Response), aSMA-Ki67 (n = 92; 49 Response, 43 Non-Response), CD3-CD31 (n = 128; 45 Response, 83 Non-Response), CD8-Ki67 (n = 249; 118 Response, 131 Non-Response), collagen–HA (n = 123; 22 Response, 101 Non-Response), granzyme B (GrzmB)-CD8 (n = 290; 137 Response, 153 Non-Response), HMGB1 (n = 147; 56 Response, 91 Non-Response), and hypoxia (n = 374; 291 Response, 83 Non-Response).

### 2.2. Sample Size, Randomisation, Masking, and Inclusion Criteria

This retrospective image-analysis study used all eligible images available from the source experimental datasets. No prospective sample-size calculation was performed for the computational analysis; sample size was determined by the availability of fluorescence images with response labels and, for the paired-animal-level fusion analysis, by the availability of matched hypoxia and collagen–HA images from the same animals. Accordingly, no effect-size-based or statistical power calculation was used to set a target sample size. The analytical sample sizes were fixed by the number of eligible labelled images available for the nine-staining benchmark (1696 images), the number of available collagen–HA images for the decomposition analysis (n = 346), and the number of animals with matched hypoxia and collagen–HA data for the paired fusion analysis (n = 104). All eligible observations were included, and no observations were sampled to reach a predetermined target. Treatment allocation and tissue generation followed the original preclinical experimental protocols. For the present retrospective computational analysis, image preprocessing, feature extraction, and model training were performed using predefined automated pipelines. Images were included if they had an assignable staining panel, response label, and usable RGB fluorescence channels. Images or animals were excluded from the paired fusion analysis only when matched hypoxia and collagen–HA data were unavailable or file identifiers could not be reconciled. The image was the unit of analysis in the nine-staining benchmark and collagen–HA decomposition analyses; the independent animal was the unit of analysis in the paired-animal-level fusion analysis. All analysed animals were female BALB/cOlaHsd mice, as detailed in Section 2.3. For the image-level benchmark and decomposition analyses, the exact numbers of animals, tissue sections per tumour, and microscopic fields per section could not be reconstructed consistently from the retrospective files. Image counts therefore describe computational observations and should not be interpreted as equivalent to independent biological replicates.

For clarity, the paired-animal-level fusion analysis was a complete-case, same-animal integration of two microscopy acquisitions. Each retained animal contributed one collagen–HA image, from which collagen and HA features were derived from the red and green channels, respectively, and one separately acquired pimonidazole/hypoxia image matched by the animal identifier. These three feature blocks were concatenated at the animal level so that each row represented one independent animal. Animals were included only when both image types and a reconciled identifier were available; animals with a missing image or unresolved identifier were excluded. The seven feature sets compared in this analysis are specified in Section 2.12. Related orthotopic tissue-generation and imaging procedures from the laboratory are described in [39], although that publication represents a separate project.

Response status was inherited from the source experimental records and was not inferred from image appearance. In the source response framework, relative tumour-volume change was calculated as the ratio of tumour volume at the end of treatment to tumour volume at the initiation of chemo-immunotherapy: values below 1.2 were classified as responsive, values between 1.2 and 2.0 as stable, and values above 2.0 as non-responsive [40]. The present computational analysis used the binary Response and Non-Response labels supplied with the image files; no labels were reassigned during preprocessing or modelling. Stable tumours were excluded before modelling, so the analysed endpoint contained responders and non-responders only. The same response thresholds were applied across treatment groups. Control animals underwent the same longitudinal monitoring and classification procedure but received placebo rather than active therapy. Absolute tumour volume at tissue collection and exact tissue-harvest timing linked to each image could not be recovered and were not included as modelling covariates.

### 2.3. Animal Model, Orthotopic Tumour Establishment, and Experimental Conditions

All tissues were derived from syngeneic orthotopic 4T1 mammary-tumour experiments. Briefly, 5 × 10^4^ 4T1 cells in 40 μL serum-free medium were implanted into the third mammary fat pad of 8-week-old female BALB/cOlaHsd mice. Animals were maintained under specific-pathogen-free conditions in the animal facilities of the Cyprus Institute of Neurology and Genetics at 22 °C and 55% relative humidity under a 12 h light–dark cycle, with unrestricted access to food and water. Tumour dimensions were monitored longitudinally using digital callipers. Control animals underwent the same monitoring and endpoint-classification workflow as treated animals but received placebo rather than active treatment. Related orthotopic 4T1 procedures have been described in previous work from the laboratory [39]; however, those publications represented separate projects conducted under their respective approvals. The tissues analysed here were generated under licence CY/EXP/PR.L01/2024.

### 2.4. Animal Ethics and ARRIVE-Related Reporting

The studies from which the datasets were derived were conducted in accordance with the animal welfare regulations and guidelines of the European Union (European Directive 2010/63/EU) and Cyprus legislation for the protection and welfare of animals. The animal study protocol was approved by the Cyprus Veterinary Services committee, the Cyprus national authority for monitoring the welfare of animals in research, under licence number CY/EXP/PR.L01/2024 (approval year: 2024).

The reporting of animal-derived data was reviewed against ARRIVE 2.0 principles [41], and a completed ARRIVE 2.0 checklist is provided as Supplementary Checklist S3. The ARRIVE 2.0 checklist is completed as a reporting map; items that were not available from the retrospective computational dataset or source experimental records are marked as not available or not reported.

### 2.5. Immunofluorescence Imaging and Image Acquisition

The analysis used RGB fluorescence immunohistochemistry images corresponding to the nine staining panels listed above. For collagen–HA images, collagen I was represented in the red channel, hyaluronic acid (HA) in the green channel, and DAPI nuclear staining in the blue channel. Fluorescence images were acquired using an Olympus BX53 fluorescence microscope at 10× magnification, unless otherwise stated. Images of each staining were acquired using identical microscope and camera settings to enable quantitative comparison across samples. Images from the tumour interior and periphery were included in the analysis. Region-specific labels were not consistently available and could not be incorporated as modelling variables; the reported results therefore represent pooled-field analyses. The acquired images were subsequently analysed using custom and built-in algorithms implemented in MATLAB [v.26.1] (MathWorks, Natick, MA, USA). Validated necrosis annotations were unavailable, and necrotic or acellular areas were not separately segmented or excluded. The complete eligible fields supplied by the source experiments were processed consistently; consequently, treatment-damage-associated tissue may contribute to the measured response signal.

The protein-marker panels were generated by fluorescence antibody-based detection. After fixation and section preparation, sections were blocked, incubated with target-specific primary antibodies, washed, and exposed to species-appropriate fluorophore-conjugated secondary antibodies; biotinylated primary reagents were visualised with fluorescent streptavidin where applicable. Thus, aPDL1 detected programmed death ligand 1; alpha-smooth muscle actin (aSMA)-CD31 combined a myofibroblast/cancer-associated-fibroblast marker with an endothelial marker; aSMA-Ki67 combined stromal activation with proliferation; CD3-CD31 combined T-cell infiltration with vasculature; CD8-Ki67 combined cytotoxic T-cell abundance with proliferation; GrzmB-CD8 combined granzyme B with cytotoxic T cells; and HMGB1 detected the damage-associated nuclear protein high-mobility group box 1. DAPI provided nuclear counterstaining. Representative source protocols used anti-collagen I, anti-CD31, anti-aSMA, and biotinylated Ki67 reagents followed by Alexa Fluor-labelled secondary antibodies or streptavidin conjugates [39]. These dual-marker combinations were analysed as co-staining panels representing complementary tissue compartments; cross-channel features measured spatial relationships within a field and were not interpreted as evidence of same-cell co-expression or direct molecular interaction.

Hyaluronan was not treated as a conventional protein antigen. In collagen–HA panels, collagen I was detected with a collagen I-specific antibody, whereas HA was localised using biotinylated hyaluronan-binding protein (bHABP), an affinity probe that binds the HA polymer and is visualised with fluorescent streptavidin [42,43]. This distinction is important because HA is a non-immunogenic glycosaminoglycan and specific anti-HA antibodies are not considered reliable for tissue localisation [43]. Hypoxia was detected using pimonidazole, a 2-nitroimidazole administered before tissue collection; under low-oxygen conditions it is reductively activated and forms stable thiol adducts that are subsequently detected with an anti-pimonidazole antibody [44]. The pimonidazole-adduct signal therefore provides a spatial readout of tissue hypoxia rather than staining a single endogenous hypoxia protein.

### 2.6. Image Preprocessing and Python Feature-Extraction Algorithm

We developed a Python [v.3.14.7] image-analysis algorithm to convert fluorescence immunohistochemistry images into reproducible quantitative histological features. Images were split into red, green, and blue channels. Each channel was thresholded using Otsu thresholding, binary masks were generated, and connected components were quantified using scikit-image region measurements [45]. The algorithm extracted 117 features per image: 18 per-channel features across three RGB channels, including intensity statistics, object count, object area descriptors, coefficient of variation of object area, and spatial spread; 22 cross-channel features for each of the three channel pairs, including overlap, Jaccard index, Pearson correlation, Manders-style co-localisation, proximity indices, and nearest-neighbour distances; six fraction-ratio features; and three global spatial features. Feature definitions are provided in Table S4. The coefficient-of-variation, intensity-distribution, object-morphology, and spatial features quantify heterogeneity within an individual microscopic field. They do not quantify between-field, between-section, or whole-tumour heterogeneity because complete field-to-section-to-animal mappings were unavailable across all staining panels.

### 2.7. Multimodal Modelling Architecture

**Figure 2:**
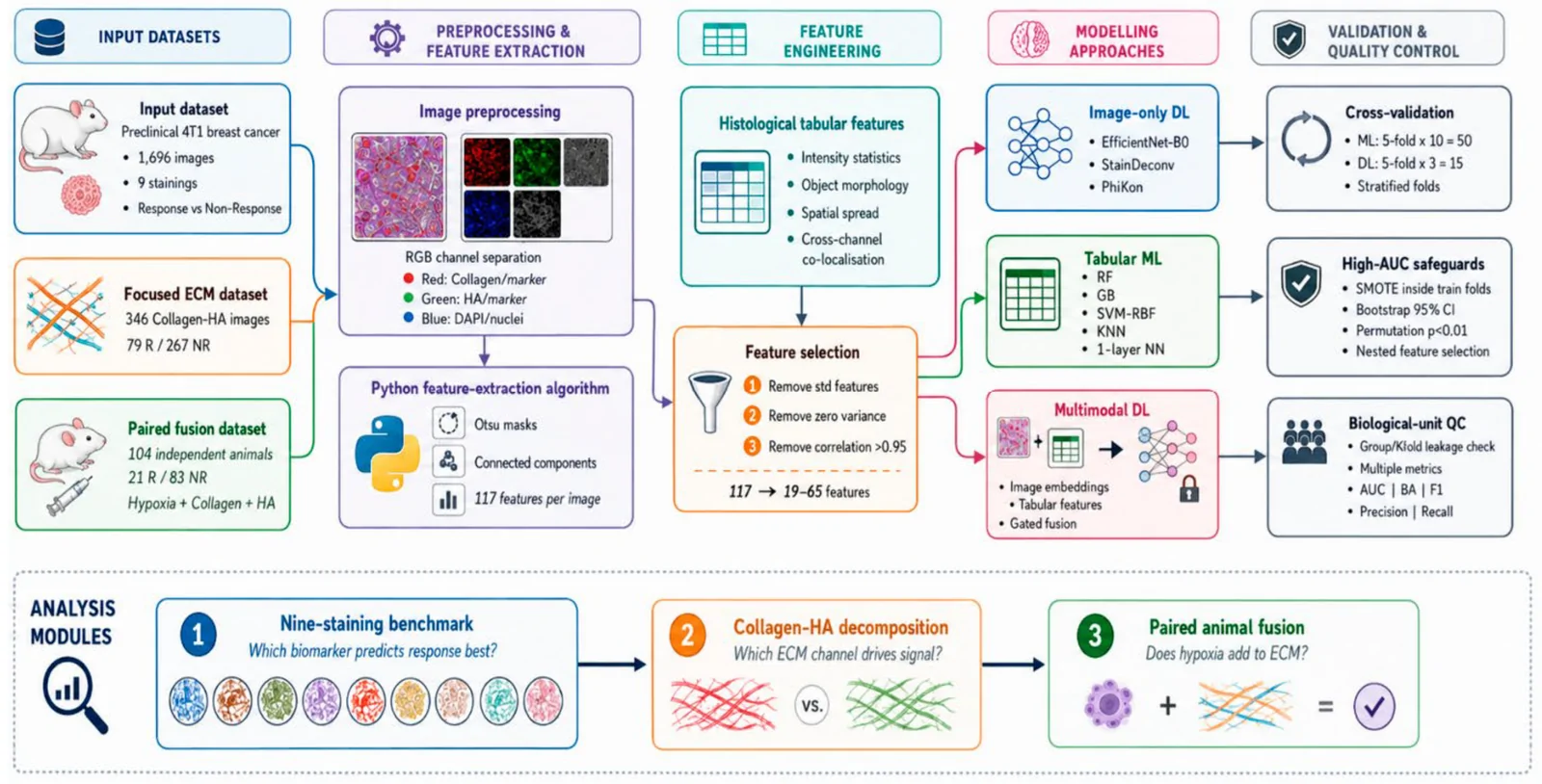
Multimodal computational pathology architecture. The workflow begins with 1696 4T1 preclinical breast cancer immunohistochemistry images across nine staining panels and proceeds through RGB-channel parsing, Python-based feature extraction, three-stage feature selection, image-only deep learning (DL), tabular machine learning (ML), and image-plus-tabular multimodal DL. Quality-control safeguards include train-fold-only Synthetic Minority Oversampling Technique (SMOTE), bootstrap 95% confidence intervals (CI), permutation testing, nested feature-selection cross-validation, GroupKFold leakage analysis, calibration, and feature-stability assessment. Generated using GPT-Image 2.0.

The modelling framework had three branches (Figure 2). First, image-only DL used raw or stain-normalised image inputs. Second, tabular ML used the 117 extracted histological features after feature selection. Third, multimodal DL fused image embeddings with tabular features through a gated fusion layer. Classical ML models were Random Forest (RF), Gradient Boosting (GB), Support Vector Machine (SVM) with radial-basis-function kernel, k-Nearest Neighbours (KNN), and a one-layer neural network (NN). DL models were EfficientNet image-only, EfficientNet plus tabular gated fusion, stain-deconvolution image-only, stain-deconvolution plus tabular gated fusion, and Phikon-v2 foundation-model representation [35,36,38,46].

### 2.8. Feature Selection and Class Imbalance

Feature selection followed three stages: removal of standard-deviation features considered redundant with coefficient-of-variation features, removal of zero-variance features, and removal of highly correlated features with Pearson correlation coefficient greater than 0.95. Depending on staining, this reduced the feature set from 117 to 19–65 features. Class imbalance was managed by stratified fold assignment, Synthetic Minority Oversampling Technique applied only inside training folds, class-weighted models where supported, and reporting of balanced accuracy, F1 score, precision, and recall in addition to AUC [47].

### 2.9. Validation and High-AUC Quality Control

Model evaluation and statistical reporting were stratified by analysis [48]. In the nine-staining benchmark, classical ML models were evaluated using five-fold cross-validation repeated ten times, yielding 50 evaluations, and DL models were evaluated using five-fold cross-validation repeated three times, yielding 15 evaluations. Benchmark DL AUCs are reported as point estimates from the original image-based and multimodal DL pipeline. Full AUC matrices for the classical ML and DL benchmark are provided in Supplementary Tables S1 and S2, respectively; model hyperparameters and fold configuration are provided in Supplementary Table S3. For the focused collagen–HA decomposition and paired-animal-level fusion analyses, bootstrap resampling was used to calculate 95% confidence intervals (CIs) from out-of-fold (OOF) predictions, and permutation testing re-ran the modelling pipeline after label shuffling 100 times, with empirical *p*-values calculated as the fraction of shuffled-label AUCs greater than or equal to the observed AUC. GroupKFold by C-code or animal identifier tested whether paired or related samples leaked across folds. Calibration plots, feature-stability bootstrapping, effect-size analysis, and DeLong-style bootstrap AUC comparisons were generated as exploratory quality-control analyses when the relevant OOF predictions were available.

### 2.10. Blinded Human-Expert Assessment

A blinded human-expert assessment was performed as an additional visual benchmark for the collagen–HA signal. Dr. Chrysovalantis Voutouri, an investigator with expertise in tumour-microenvironment biology, cancer biomechanics, and computational cancer research, assessed 100 collagen–HA fluorescence images, comprising 50 responder and 50 non-responder images. Images were randomly coded, and the assessor was blinded to response labels and treatment groups. Each image was classified as responder or non-responder. Performance was summarised using the confusion matrix, sensitivity, specificity, F1 score, and balanced accuracy. Because the expert generated binary decisions rather than continuous probability scores, the single-threshold receiver-operating-characteristic AUC is equivalent to balanced accuracy. The 95% confidence interval was taken from the original expert-assessment analysis.

### 2.11. Computational Environment and Software

All image processing, feature extraction, model training, statistical validation, and figure generation were performed in Python 3.11.15. The main analysis was developed using NumPy 2.4.6, pandas 3.0.3, scikit-image 0.26.0, scikit-learn 1.8.0, imbalanced-learn 0.14.1, SciPy 1.17.1, matplotlib 3.10.9, seaborn 0.13.2, PyTorch 2.12.0, torchvision 0.27.0, and timm 1.0.27. Classical ML models, cross-validation routines, feature scaling, permutation testing, and metric calculations were implemented with scikit-learn [49]. DL models were implemented in PyTorch and torchvision, with pretrained EfficientNet-B0 and related image encoders loaded through torchvision or timm where applicable. Tabular data handling and result aggregation were performed with pandas and NumPy. Statistical tests, bootstrap resampling, and confidence-interval calculations used NumPy, SciPy, and scikit-learn utilities. The complete computational environment, including package versions, random seeds, model hyperparameters, and cross-validation settings, is reported in Table S3.

### 2.12. Experimental Modules

Three analyses were performed. The nine-staining benchmark compared the best model for each staining panel. The collagen–HA decomposition analysis used 346 images (79 Response, 267 Non-Response) to compare collagen-only red-channel features, HA-only green-channel features, combined collagen-plus-HA features, and cross-channel interaction-only features. Interaction-only features were defined as features that quantify the spatial relationship between collagen and HA signals, including pixel-level overlap, co-localisation, intensity correlation, and nearest-neighbour proximity between the two channels.

The purpose of the paired-animal-level fusion analysis was to test whether hypoxia provided predictive information beyond ECM features in the same biological units. We therefore included only animals with matched hypoxia and collagen–HA staining data. Each animal, rather than each image, was treated as one independent observation. Collagen and HA features were extracted from the red and green channels of the same collagen–HA image, and hypoxia features were extracted from a separate hypoxia image matched to the same animal. The final paired dataset contained 104 animals, including 21 responders and 83 non-responders. Seven feature sets were compared: hypoxia-only, collagen-only, HA-only, collagen-plus-HA, hypoxia-plus-collagen, hypoxia-plus-HA, and hypoxia-plus-collagen-plus-HA.

### 2.13. Reporting Standards

This manuscript was prepared with reference to ARRIVE 2.0 for animal-derived data, TRIPOD/TRIPOD+AI for prediction-model reporting, and CLAIM for medical-imaging artificial intelligence reporting where applicable [41,48,50,51,52]. Completed ARRIVE 2.0, TRIPOD+AI, and CLAIM checklist files are provided as Supplementary Checklists S1–S3. Because this was a retrospective secondary computational analysis of preclinical images, some ARRIVE acquisition, husbandry, and experimental-allocation details were not available in the computational dataset or source experimental records and are marked in the ARRIVE checklist as not available or not reported.

## 3. Results

### 3.1. Dataset and Workflow

The study analysed 1696 immunohistochemistry images from preclinical 4T1 breast cancer therapy-response datasets across nine staining panels (Figure 1). The stainings captured immune-checkpoint, immune-cell, proliferation, vascular, stromal, ECM, cytotoxicity, damage-associated, and hypoxia-associated biological markers. Image features were extracted from RGB intensity channels, thresholded binary masks, connected components, and cross-channel spatial relationships. After feature selection, each staining retained 19–65 features for classical ML evaluation. DL models used image inputs alone or image features fused with tabular handcrafted features.

At the animal level, treatment composition differed markedly between response classes. Responders comprised mechanotherapeutic plus chemotherapy (20%), mechanotherapeutic plus immune-checkpoint inhibition (27%), and mechanotherapeutic plus chemotherapy plus immune-checkpoint inhibition (53%). Non-responders comprised chemotherapy (36%), immune-checkpoint inhibition (31%), chemotherapy plus immune-checkpoint inhibition (25%), and mechanotherapeutic plus chemotherapy (8%). These percentages describe the distribution of treatment groups within each response class, not the response rate within a treatment arm. The association between treatment group and response class is therefore a potential source of confounding in the pooled cross-treatment analyses.

### 3.2. Histological Staining Panels Classified Treatment-Response Status Across the Nine-Panel Benchmark

**Figure 3:**
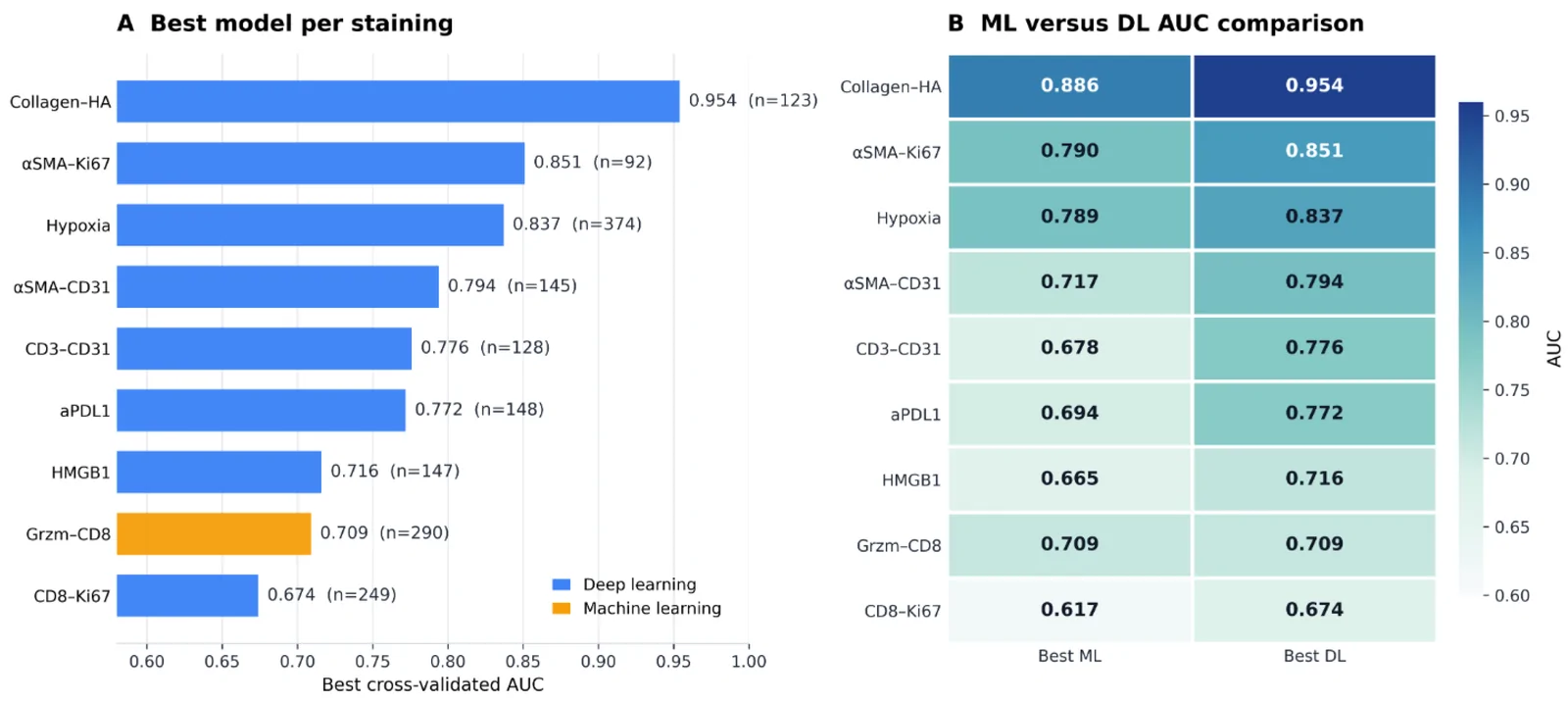
Nine histological staining-panel benchmarks for treatment-response classification. (**A**) Ranked best cross-validated area under the receiver-operating-characteristic curve (AUC) per staining. (**B**) Comparison between the best tabular machine learning (ML) AUC and the best deep learning (DL) AUC for each staining. Classical ML models used repeated stratified cross-validation; DL models used repeated stratified cross-validation with image-only or image-plus-tabular inputs. N values denote image counts per staining. Bars and the heatmap show the highest cross-validated AUC available for each staining and modelling paradigm. Generated using Python [v.3.14.7].

The nine-staining benchmark tested whether histological staining panels captured by immunofluorescence imaging could classify treatment-response status in the 4T1 breast cancer model (Figure 3; Tables S1 and S2). Across markers of immune-checkpoint signalling, vascular and stromal structure, proliferation, immune infiltration, cytotoxic activity, tissue damage, ECM, and hypoxia, several stainings showed discriminatory performance, supporting the central premise that treatment response is encoded in tissue architecture and biomarker distribution. The strongest signal was ECM-related: collagen–HA was the highest-performing co-staining, with the best AUC equal to 0.954 using an EfficientNet image-only model. This exceeded the next-best marker pair, alpha-smooth muscle actin-Ki67, which achieved an AUC of 0.851 using stain-deconvolution plus tabular fusion, and hypoxia, which ranked third with AUC equal to 0.837 using EfficientNet image-only classification.

As a secondary methodological finding, DL achieved the best AUC in eight out of nine histological markers, indicating that image-based models frequently captured predictive spatial and textural information beyond tabular handcrafted features. The GrzmB-CD8 pair was the only staining in which a classical ML model was not inferior to DL models (AUC 0.709). Because the benchmark combined classical ML and DL pipelines with different output structures, the DL benchmark AUCs are reported as point estimates from the original pipeline; full benchmark AUC matrices for ML and DL models are shown in Tables S1 and S2. For the collagen–HA decomposition and paired-animal-level fusion analyses, complete bootstrap 95% CI, permutation *p* values, balanced accuracy, F1 score, precision, and recall are reported in Table 1.

### 3.3. Collagen–HA Decomposition Identified Collagen as the Stronger Single-Channel Predictor in the Full Dataset

**Figure 4:**
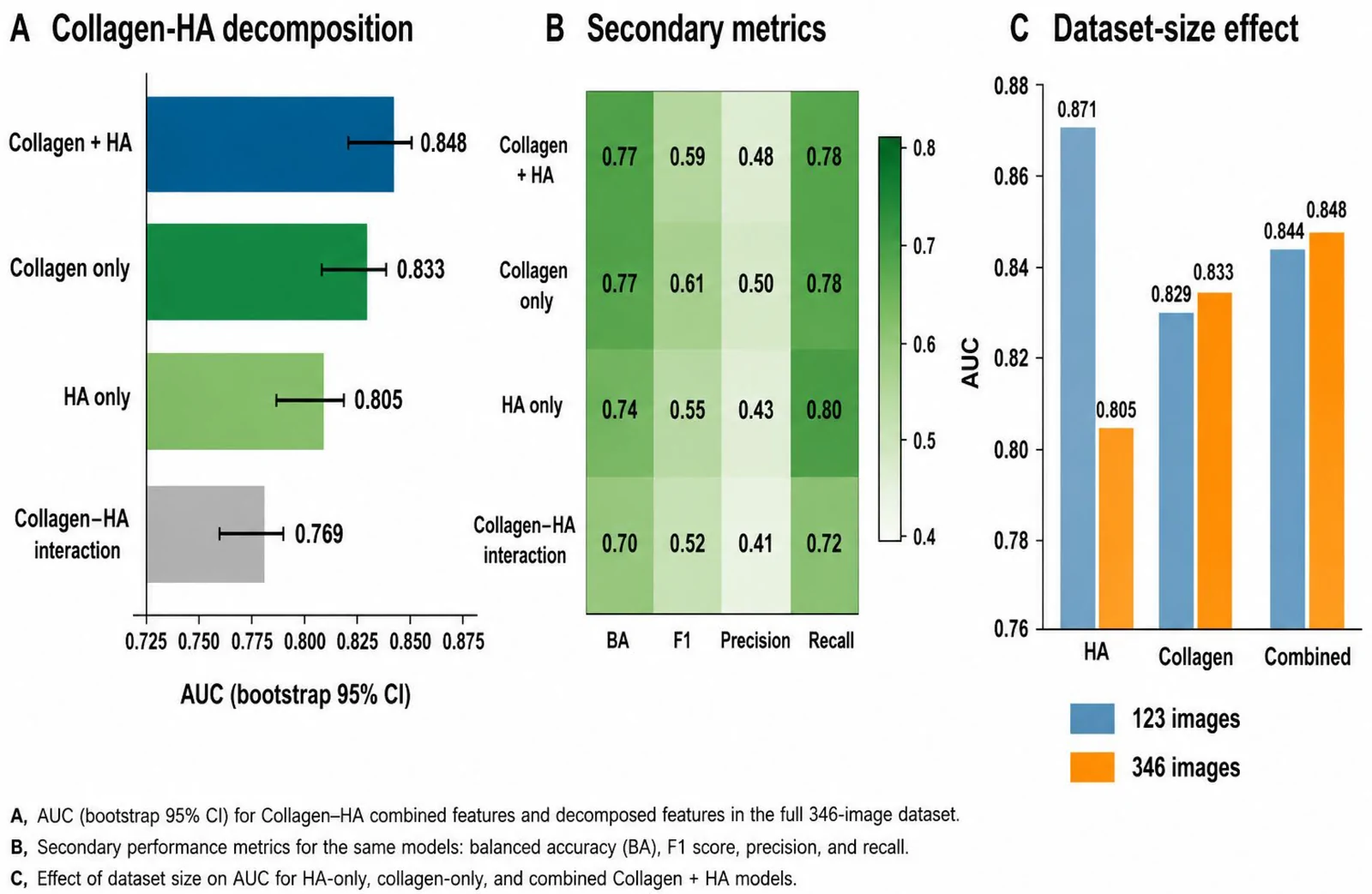
Collagen–HA decomposition in 346 images. (**A**) AUC comparison for collagen-only (dark green), HA-only (light green), combined collagen-plus-HA (blue), and interaction-only feature configurations (grey). Error bars show bootstrap 95% confidence intervals. (**B**) Balanced accuracy (BA), F1 score, precision, and recall for each feature configuration (i.e., darker green indicates higher metric scores). (**C**) Comparison of the 123-image subset (light blue) and the 346-image (orange) full dataset, showing the reversal from hyaluronic-acid dominance in the smaller subset to collagen dominance in the full dataset. Generated using Python [v.3.14.7].

Because collagen–HA was the best-performing co-staining panel in the nine-staining benchmark, we decomposed the dual fluorescence images into biologically defined marker channels (Figure 4). In the original 123-image benchmark subset, HA-only features had numerically outperformed collagen-only features (AUC 0.871 vs. 0.829), suggesting that HA contributed more strongly to the collagen–HA signal in that smaller dataset. We therefore repeated the decomposition analysis in the expanded 346-image collagen–HA dataset, which included 79 Response and 267 Non-Response images. In this larger dataset, the relative ranking changed: collagen-only features achieved higher discrimination than HA-only features. Combined collagen-plus-HA features achieved the highest performance, with AUC 0.848 (95% CI 0.832–0.861; *p* < 0.01), balanced accuracy 0.765, F1 score 0.595, precision 0.485, and recall 0.778. Collagen-only features achieved AUC 0.833 (95% CI 0.817–0.847; *p* < 0.01), while HA-only features achieved AUC 0.805 (95% CI 0.784–0.814; *p* < 0.01). Cross-channel interaction-only features, defined as features quantifying the spatial relationship between collagen and HA signals rather than either marker alone, including overlap, co-localisation, correlation, and proximity, were weakest, with AUC 0.769 (95% CI 0.748–0.781; *p* < 0.01).

Blinded human assessment of 100 collagen–HA images yielded 41 true positives, 43 true negatives, 7 false positives, and 9 false negatives. Sensitivity was 0.82, specificity was 0.86, F1 score was 0.84, and balanced accuracy/single-threshold AUC was 0.84 (95% CI 0.74–0.88). These findings provide a blinded visual benchmark and indicate that response-associated ECM morphology was detectable by expert review. The expert AUC was below the reported computational AUCs, although the differing evaluation designs preclude a formal superiority comparison.

### 3.4. Paired-Animal-Level Fusion Showed That ECM Markers Outperform Hypoxia

**Figure 5:**
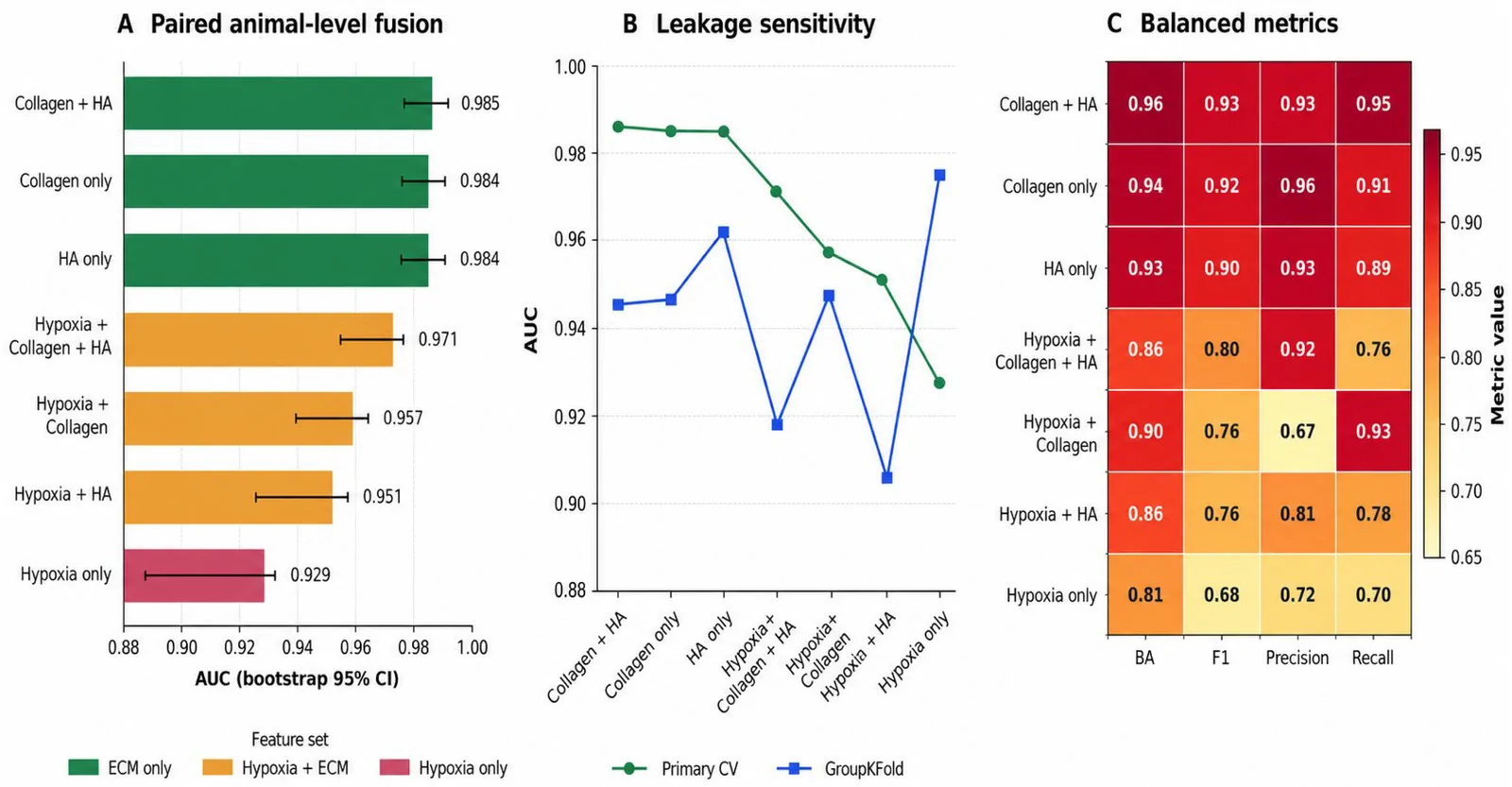
Animal-level multi-staining fusion in 104 paired animals. (**A**) AUC comparison across seven paired-animal-level experiments. Error bars show bootstrap 95% confidence intervals. The feature-set legend is placed outside the bar chart. (**B**) Leakage-sensitivity analysis comparing primary cross-validation and GroupKFold by C-code or animal identifier. (**C**) Balanced accuracy, F1 score, precision, and recall for each paired-animal-level feature configuration. ECM, extracellular matrix; HA, hyaluronic acid. Generated using Python [v.3.14.7].

The paired-animal-level analysis included 104 independent animals, of which 21 were responders and 83 were non-responders. Each animal contributed paired hypoxia and collagen–HA information. Collagen plus HA achieved AUC 0.985 (95% CI 0.981–0.992; *p* < 0.01), balanced accuracy 0.962, F1 score 0.928, precision 0.926, and recall 0.953 (Figure 5). HA alone achieved AUC 0.984 (95% CI 0.974–0.991; *p* < 0.01), and collagen alone achieved AUC 0.984 (95% CI 0.977–0.991; *p* < 0.01). Hypoxia alone was predictive but weaker than ECM features, with AUC 0.929 (95% CI 0.887–0.931; *p* < 0.01). Adding hypoxia to ECM features did not improve performance.

### 3.5. Validation Analyses Supported Robustness

**Figure 6:**
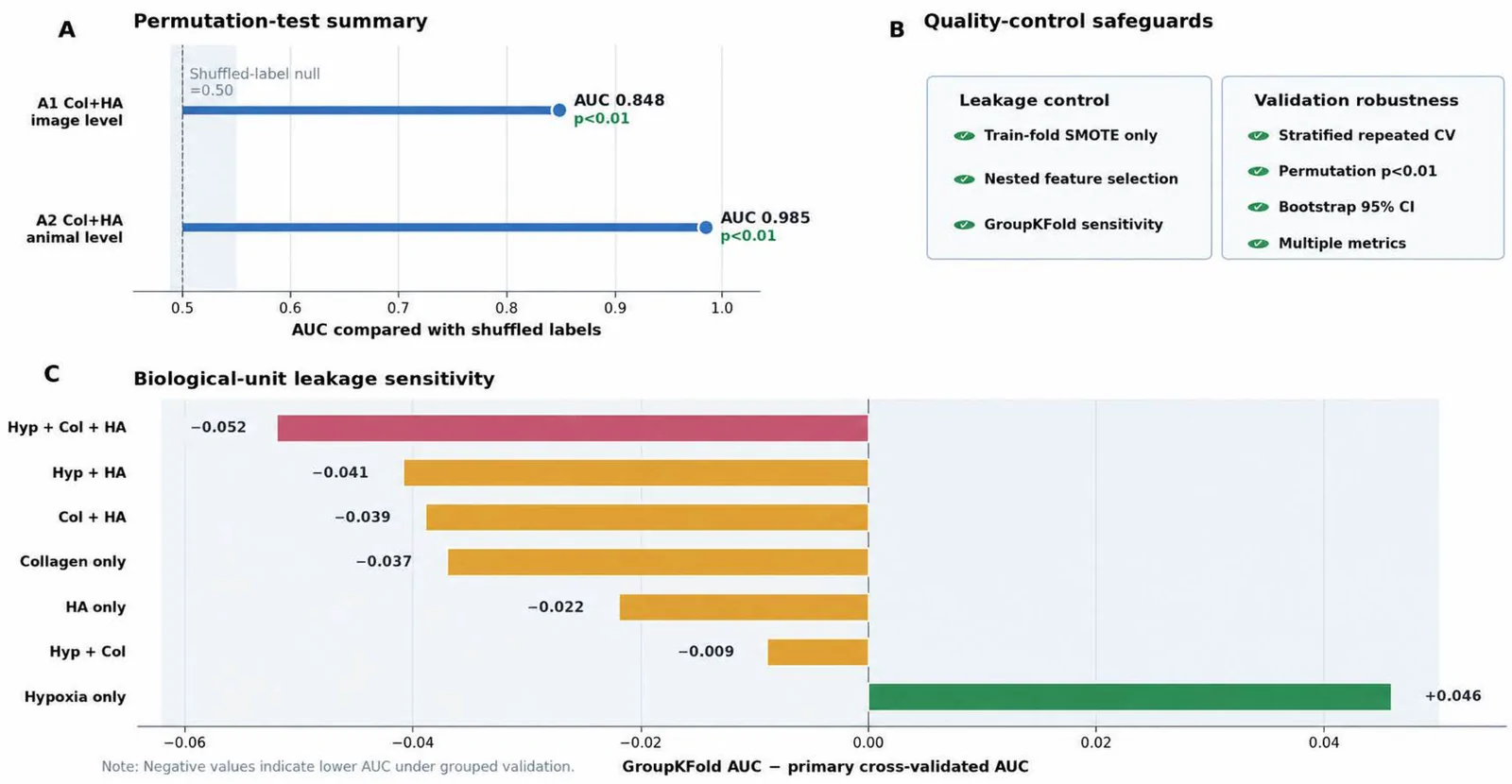
Statistical quality-control and high-AUC validation. (**A**) Permutation-test summary showing observed AUCs for the main ECM models relative to the shuffled-label null. (**B**) Summary of safeguards used to reduce artefactual performance inflation. (**C**) Difference between GroupKFold AUC and primary AUC for paired-animal-level experiments. Negative values indicate lower AUC under grouped validation. AUC, area under the receiver-operating-characteristic curve; CI, confidence interval; CV, cross-validation; ECM, extracellular matrix; HA, hyaluronic acid; SMOTE, Synthetic Minority Oversampling Technique. Panels A and C were generated using Python [v.3.14.7]. Panel B generated GPT-Image 2.0. AUC = area under the receiver-operating-characteristic curve; BA = balanced accuracy; DL = deep learning; GB = Gradient Boosting; HA = hyaluronic acid; Hyp = hypoxia; KNN = k-Nearest Neighbours; ML = machine learning; RF = Random Forest; SVM = Support Vector Machine. For the nine-staining benchmark, the best overall AUC reports the best model from the original ML/DL benchmark. DL benchmark AUCs are point estimates from the original pipeline. Dashes indicate that bootstrap confidence intervals, permutation *p* values, and secondary metrics were not generated for those benchmark point estimates and were not imputed. Full ML and DL benchmark AUC matrices are provided in Tables S1 and S2. For decomposition and animal-level fusion analyses, all reported 95% confidence intervals, *p* values, BA, F1, precision, and recall correspond to the listed best model.

Permutation testing showed that all experiments across the decomposition and fusion analyses performed above chance, with empirical *p* < 0.01 and null AUC distributions centred near 0.50 (Figure 6). Learning curves from the nine-staining biomarker benchmark showed that seven of nine pairs were still improving with additional training data, whereas GrzmB-CD8 and CD8-Ki67 appeared closer to saturation (Figure S1). GroupKFold by C-code showed no severe leakage effect: the maximum AUC reduction was 0.052, and all grouped AUCs remained above 0.91. The conservative group composition used for the leakage-sensitivity analysis is shown in Figure S2. The high AUCs are therefore unlikely to be explained solely by class imbalance, threshold choice, feature-selection leakage, or paired-sample leakage. Nevertheless, the absence of external validation remains an important limitation. Grouped validation was possible only where C-code or animal identifiers were available and does not eliminate possible field-level pseudoreplication in the full nine-panel benchmark, for which complete animal and section mappings were unavailable.

## 4. Discussion

This study shows that quantitative immunohistochemistry contains a strong and biologically coherent signal for treatment-response classification in a preclinical breast cancer model. Across nine staining panels and ten machine learning (ML) or deep learning (DL) models, the strongest single-staining result came from collagen–HA, which achieved an area under the receiver-operating-characteristic curve (AUC) of 0.954 using EfficientNet image-only classification. DL achieved the best AUC in eight of nine staining panels, supporting the value of spatial and textural image representations beyond summary features. Focused decomposition of the full 346-image collagen–HA dataset showed that collagen was the stronger single channel, while combined collagen and HA features performed best overall. In the paired-animal-level analysis, extracellular-matrix (ECM) markers achieved an AUC of 0.985, outperformed hypoxia, and were not improved by adding hypoxia.

These findings are also consistent with preclinical breast cancer studies from the same laboratory, in which pharmacological normalisation of the tumour microenvironment reduced collagen and HA abundance, improved vascular perfusion, increased intratumoural T-cell infiltration, and enhanced chemo-immunotherapy efficacy [39]. More broadly, the observed predictive value of ECM architecture is supported by published studies linking stromal organisation, collagen architecture, HA remodelling, and collagen-mediated immune exclusion to breast cancer treatment response [53,54,55,56,57,58,59].

The predominance of the stromal signal is consistent with published breast cancer studies, although the biological systems, treatment regimens, and response endpoints differ. Farmer et al. identified a stroma-related gene-expression signature associated with resistance to anthracycline-containing neoadjuvant chemotherapy [53]. In the NEOZOTAC trial, disorganised stroma in pretreatment biopsies was associated with a poorer response to neoadjuvant chemotherapy [54], while the tumour–stroma ratio was associated with Miller–Payne score and pathological response in HER2-negative early breast cancer [55]. More recently, Li et al. developed a DL-derived tumour-associated stroma score that independently predicted pathological complete response in a multicentre cohort; collagen- and fibroblast-dominant stromal patterns were associated with poorer response, whereas lymphocyte-rich patterns were associated with greater benefit [56]. The present results extend this general evidence into a controlled preclinical multi-staining setting by indicating that ECM architecture can carry more predictive information than several immune, vascular, proliferation, damage-associated, and hypoxia-related stainings.

The collagen-specific result also agrees with imaging studies showing that collagen organisation contains treatment-relevant information. Using second-harmonic-generation microscopy in breast cancer core biopsies, Desa et al. reported that collagen directionality was associated with neoadjuvant chemotherapy response in HER2-positive disease but not in triple-negative breast cancer [57]. That subtype-dependent finding cautions against treating collagen as a uniform biomarker and suggests that alignment, topology, and spatial context may be as important as abundance. In the present study, collagen-only features outperformed HA-only features in the expanded dataset, while combined collagen-plus-HA features were strongest. Conversely, cross-channel interaction-only features were weaker, suggesting that marker-specific burden, heterogeneity, morphology, and spatial distribution contributed more information than co-localisation alone. Differences from the human biopsy literature are expected because this analysis used a 4T1 model, mixed treatment classes, fluorescence-derived channels, and source-defined response rather than pathological complete response.

The combined collagen–HA signal is biologically plausible because both matrix components influence tumour mechanics, transport, and immune access [15,16,17,18,19,20,21,22,23,24]. In experimental breast tumours, remodelling a hyaluronan-rich microenvironment sensitised tumours to anti-programmed death ligand 1 immunotherapy [58]. Separately, tumour discoidin domain receptor 1 was shown to promote collagen–fibre alignment and immune exclusion, linking collagen architecture directly to impaired T-cell access [59]. Together with evidence that collagen density suppresses tumour-infiltrating T-cell activity [5] and that microenvironment-normalisation strategies can improve immunotherapy [24], these studies support the interpretation that the present imaging features capture biologically meaningful stromal states. Nevertheless, the current analysis is predictive rather than mechanistic and cannot establish whether collagen or HA directly caused treatment resistance in the analysed animals.

The hypoxia findings should be interpreted within the same mechanistic framework. Hypoxia alone was strongly predictive at animal level, with an AUC of 0.929, but adding hypoxia to ECM features reduced rather than improved discrimination. A dense or swollen matrix can compress vessels, reduce perfusion, limit drug transport, and generate hypoxic compartments [19,20,21,22,23,24,26,27,28]. Consequently, part of the hypoxia-associated signal may already be encoded by collagen and HA morphology. The reduced performance of combined feature sets may therefore reflect biological redundancy, finite paired-sample size, increased model dimensionality, or treatment-specific interactions rather than a lack of importance of hypoxia. Spatially registered analyses are required to determine whether ECM and hypoxia co-localise, represent sequential states, or identify distinct microenvironmental phenotypes.

The computational pathology results are also consistent with prior response-prediction studies in human breast cancer. Ali et al. showed that computationally quantified lymphocyte density in pretreatment biopsies predicted response to neoadjuvant chemotherapy [60]. Saednia et al. combined quantitative nuclear morphology, intensity, texture, graph, and wavelet features with ML and achieved an independent-test AUC of 0.90 in 149 patients [61]. Duanmu et al. integrated serial haematoxylin-and-eosin, Ki67, and phosphohistone H3 images using spatial-attention DL to predict pathological complete response in triple-negative breast cancer [62]. These studies support the principle that response information is distributed across tissue architecture and that automated image analysis can recover information not captured by conventional visual scoring. The present study differs by benchmarking nine fluorescence staining panels, comparing image-only DL with interpretable tabular features, and decomposing the dominant ECM signal at both the image and paired-animal levels.

The magnitude of the present AUCs requires particular caution when compared with human studies. The PROACTING study evaluated DL biomarkers derived from routine diagnostic biopsies across breast cancer subtypes and reported performance that varied substantially between development and evaluation cohorts [63]. A 2025 multicentre whole-slide study similarly reported an internal response-prediction AUC of 0.869 and a pooled external AUC of 0.841 [64]. Most recently, the 2026 TIGER challenge analysis benchmarked computational tumour-infiltrating-lymphocyte models in a large multicentre breast cancer dataset and evaluated associations with pathological complete response and survival [65]. These published results reinforce the value of tissue-based computational biomarkers, while also showing the importance of sequestered, multicentre, and independent validation. The reported human-study performance is lower than the internal AUCs of 0.954 and 0.985 reported here, but the estimates are not directly comparable. The present analysis used a preclinical model, smaller and more controlled datasets, internal repeated cross-validation, source-defined response labels, and partially imbalanced classes, whereas the human studies incorporated inter-patient heterogeneity, variable tissue acquisition, clinical subtype diversity, and external validation. The high AUCs should therefore be regarded as evidence of a strong preclinical signal rather than an estimate of expected clinical performance.

The study was designed as pooled cross-treatment response-status classification rather than therapy-specific biomarker validation. Treatment composition differed strongly between response classes, and subdivision by treatment, staining panel, and response would have produced small and highly imbalanced groups. Reliable treatment-specific modelling was therefore not performed. Treatment-associated tissue effects may consequently contribute to model performance, and the findings should not be interpreted as demonstrating a universal treatment-independent predictor.

The blinded human-expert assessment provided an additional interpretability check. Dr. Voutouri classified 100 collagen–HA images with sensitivity 0.82, specificity 0.86, F1 score 0.84, and balanced accuracy/single-threshold AUC 0.84 (95% CI 0.74–0.88). This indicates that part of the response-associated ECM phenotype was visually recognisable. However, only one expert was assessed, inter-rater reproducibility was not evaluated, and the result should not be considered equivalent to independent pathological validation.

Several limitations constrain interpretation. First, this preclinical study does not establish clinical utility in human breast cancer. Second, the paired-animal-level fusion analysis included only 104 animals, with 21 responders, and 276 collagen–HA image pairs were unavailable. Third, all primary performance estimates were derived from internal cross-validation; no independent cohort from another laboratory, microscope, staining batch, or treatment experiment was available. Fourth, the benchmark DL AUCs were point estimates from the original pipeline and did not have the same confidence-interval and permutation-test outputs as the focused analyses. Fifth, computational features have not yet been fully mapped to pathologist-validated tissue phenotypes. Sixth, some original animal-protocol and microscopy-acquisition details were unavailable in the retrospective computational dataset. Finally, treatment heterogeneity may contribute to the observed signal, and the current sample sizes did not support reliable treatment-specific stratification.

Additional design limitations affect biological interpretation. Necrotic or acellular regions were not separately segmented; tumour-interior and peripheral fields were pooled because region labels were unavailable; absolute tumour size and tissue-harvest timing linked to each image could not be recovered; and complete animal-section-field mappings were unavailable for the image-level benchmark, leaving potential pseudoreplication. The pipeline quantified within-field heterogeneity but not between-field, between-section, or whole-tumour heterogeneity. Together with the association between the treatment group and response class, these factors mean that the models should be interpreted as pooled response-status classifiers rather than treatment-independent or pathologist-validated predictors.

The next step is prospective external validation. A locked analysis pipeline should first be tested in a larger matched animal cohort with complete hypoxia and collagen–HA imaging, followed by an independent experiment performed using separate staining batches and imaging conditions. Translation should then proceed to human archival breast cancer tissue with a known treatment response, ideally using standardised multiplex immunohistochemistry or immunofluorescence and comparison with established predictors such as programmed death ligand 1, tumour-infiltrating lymphocytes, tumour–stroma ratio, stromal percentage, and molecular subtype. Pathologist annotation, second-harmonic-generation collagen imaging, multiplex immune profiling, and spatial transcriptomics would help determine whether the predictive features represent collagen alignment, HA accumulation, immune exclusion, vascular compression, matrix stiffness, or other stromal phenotypes. Such studies are necessary before quantitative ECM imaging can be considered a clinically relevant therapy-response biomarker. Future preclinical studies should additionally record treatment-specific response rates, baseline and endpoint tumour volumes, tissue-harvest timing, animal-section-field mappings, and field location; incorporate viable-tissue or necrosis segmentation; perform treatment-stratified and leave-one-treatment-group-out analyses; aggregate multi-field heterogeneity at the animal level; and include multiple blinded experts to establish inter-rater reproducibility.

## 5. Conclusions

This preclinical study identifies quantitative ECM imaging as a strong image-derived biomarker axis for treatment-response classification in 4T1 breast cancer. Collagen and HA features carried the dominant signal, outperformed hypoxia in paired animals, and were measurable using a reproducible computational pathology pipeline combining image-only DL, tabular ML, and image-plus-tabular fusion.

The findings should be interpreted as hypothesis-generating rather than clinically validated. Independent preclinical replication, full microscopy and animal-protocol reporting, raw-image availability, and validation in human archival breast cancer tissue are required before quantitative ECM imaging can be considered for clinical biomarker development.

## Figures and Tables

**Table 1:** Summary of model performance across the benchmark, decomposition, and animal-level fusion analyses.

Group	Experiment	N	Best Overall Model	Overall AUC	Best ML Model (AUC)	95% CI	*p* Value	BA	F1	Prec/Rec
Benchmark	Collagen–HA	123	EffNet (image)	0.954	RF (0.886)	—	—	—	—	—
Benchmark	aSMA-Ki67	92	StainDeconv+Tab	0.851	GB (0.790)	—	—	—	—	—
Benchmark	Hypoxia	374	EffNet (image)	0.837	SVM (0.789)	—	—	—	—	—
Benchmark	aSMA-CD31	145	EffNet+Tab	0.794	RF (0.717)	—	—	—	—	—
Benchmark	CD3-CD31	128	EffNet (image)	0.776	RF (0.678)	—	—	—	—	—
Benchmark	aPDL1	148	StainDeconv+Tab	0.772	SVM (0.694)	—	—	—	—	—
Benchmark	HMGB1	147	EffNet (image)	0.716	GB (0.665)	—	—	—	—	—
Benchmark	Grzm-CD8	290	RF	0.709	RF (0.709)	—	—	—	—	—
Benchmark	CD8-Ki67	249	EffNet (image)	0.674	KNN (0.617)	—	—	—	—	—
Decomposition	Collagen + HA	346	KNN	0.848	KNN (0.848)	0.832–0.861	<0.01	0.765	0.595	0.485/0.778
Decomposition	Collagen only	346	KNN	0.833	KNN (0.833)	0.817–0.847	<0.01	0.774	0.607	0.500/0.782
Decomposition	HA only	346	RF	0.805	RF (0.805)	0.784–0.814	<0.01	0.738	0.552	0.426/0.795
Decomposition	Col-HA interaction	346	RF	0.769	RF (0.769)	0.748–0.781	<0.01	0.703	0.517	0.406/0.718
Fusion	Col + HA	104	KNN	0.985	KNN (0.985)	0.981–0.992	<0.01	0.962	0.928	0.926/0.953
Fusion	HA only	104	KNN	0.984	KNN (0.984)	0.974–0.991	<0.01	0.933	0.895	0.927/0.894
Fusion	Collagen only	104	KNN	0.984	KNN (0.984)	0.977–0.991	<0.01	0.945	0.920	0.958/0.907
Fusion	Hyp + Col + HA	104	GB	0.971	GB (0.971)	0.952–0.974	<0.01	0.865	0.804	0.917/0.759
Fusion	Hyp + Col	104	KNN	0.957	KNN (0.957)	0.936–0.962	<0.01	0.896	0.761	0.669/0.928
Fusion	Hyp + HA	104	GB	0.951	GB (0.951)	0.925–0.959	<0.01	0.858	0.764	0.806/0.781
Fusion	Hypoxia only	104	SVM	0.929	SVM (0.929)	0.887–0.931	<0.01	0.807	0.680	0.720/0.703

## Data Availability

The datasets generated and/or analysed during the current study were obtained from in vivo experiments conducted in the authors’ laboratory and are available from the corresponding author on reasonable request, subject to institutional data-sharing requirements. The underlying code for this study is available at https://github.com/stelioslamprou37/IFImage_Based_BreastCancer_Therapy_Response (accessed 1 February 2026). Raw and unprocessed microscopy image files are available from the corresponding author on reasonable request, subject to institutional data-sharing requirements.

## Supplementary Materials

The following supporting information can be downloaded at https://www.mdpi.com/article/10.3390/cancers18162603/s1: Table S1: Classical machine learning benchmark AUCs for all five tabular models across nine stainings; Table S2: Deep learning benchmark point estimates for all five image-based or multimodal architectures across nine stainings; Table S3: Model hyperparameters, software versions, random seeds, and fold configuration; Table S4: Feature dictionary for all 117 extracted image features; Figure S1: Learning curves for all nine staining pairs; Figure S2: Conservative group composition used for GroupKFold leakage-sensitivity analysis; Supplementary Checklist S1: TRIPOD+AI reporting checklist; Supplementary Checklist S2: CLAIM 2024 reporting checklist; Supplementary Checklist S3: ARRIVE 2.0 reporting checklist. Raw and unprocessed microscopy image files are available from the corresponding author on reasonable request, subject to institutional data-sharing requirements.
