# Supplementary material for: Computational Pathology Reveals Extracellular-Matrix Imaging Biomarkers of Therapy Response in Preclinical Breast Cancer
Source: Cancers (Basel). 2026 Aug 12;18(16):2603. doi: 10.3390/cancers18162603 (PMC13510428; doi:10.3390/cancers18162603)
Supplement: Supplementary file 1 [file cancers-18-02603-s001.zip › cancers-4451134-supplementary.pdf]

Supplementary Materials

Computational Pathology Reveals Extracellular-Matrix Imaging Biomarkers of Therapy Response in Preclinical Breast Cancer

Stelios Lamprou, Styliana Georgiou, Triantafyllos Stylianopoulos and Chrysovalantis Voutouri

The following supplementary materials are provided: Supplementary Tables S1–S4, Supplementary Figures S1-S2, Supplementary Checklist S1: TRIPOD+AI reporting checklist, Supplementary Checklist S2: CLAIM 2024 reporting checklist, and Supplementary Checklist S3: ARRIVE 2.0 reporting checklist. Raw and unprocessed microscopy image files are available from the corresponding author on reasonable request, subject to institutional data-sharing requirements.

Supplementary Table S1. Classical machine learning benchmark AUCs for all five tabular models across nine stainings.

Staining	N	R/NR	Features (117→)	RF AUC	GB AUC	SVM AUC	KNN AUC	1L-NN AUC	Best ML	Best AUC
aPDL1	148	110/38	→19	0.666	0.663	0.694	0.627	0.580	SVM	0.694
aSMA-CD31	145	84/61	→59	0.717	0.705	0.680	0.659	0.610	RF	0.717
aSMA-Ki67	92	49/43	→65	0.770	0.790	0.750	0.745	0.680	GB	0.790
CD3-CD31	128	45/83	→56	0.678	0.660	0.645	0.670	0.590	RF	0.678
CD8-Ki67	249	118/131	→58	0.610	0.590	0.617	0.600	0.550	KNN	0.617
Collagen-HA	123	22/101	→34	0.886	0.850	0.840	0.870	0.720	RF	0.886
Grzm-CD8	290	137/153	→63	0.709	0.690	0.680	0.695	0.630	RF	0.709
HMGB1	147	56/91	→26	0.650	0.665	0.640	0.620	0.580	GB	0.665
Hypoxia	374	291/83	→24	0.770	0.760	0.789	0.750	0.700	SVM	0.789

Supplementary Table S2. Deep learning benchmark point estimates for all five image-based or multimodal architectures across nine stainings.

Staining	N	EffNet(img)	EffNet+Tab	StainDeconv(img)	StainDeconv+Tab	Phikon-v2	Best DL	Best AUC	DL>ML?
aPDL1	148	0.740	0.755	0.760	0.772	0.710	StainDeconv+Tab	0.772	Yes
aSMA-CD31	145	0.780	0.794	0.760	0.770	0.720	EffNet+Tab	0.794	Yes
aSMA-Ki67	92	0.830	0.840	0.845	0.851	0.790	StainDeconv+Tab	0.851	Yes
CD3-CD31	128	0.776	0.760	0.740	0.750	0.700	EffNet(img)	0.776	Yes
CD8-Ki67	249	0.674	0.660	0.650	0.640	0.620	EffNet(img)	0.674	Yes
Collagen-HA	123	0.954	0.940	0.920	0.930	0.880	EffNet(img)	0.954	Yes
Grzm-CD8	290	0.690	0.680	0.670	0.660	0.640	EffNet(img)	0.690	No (ML wins)
HMGB1	147	0.716	0.700	0.690	0.710	0.670	EffNet(img)	0.716	Yes
Hypoxia	374	0.837	0.820	0.810	0.825	0.780	EffNet(img)	0.837	Yes

Supplementary Table S3. Model hyperparameters, software versions, random seeds, and fold configuration.

Component	Parameter	Value
Random Forest	n_estimators	200
	max_depth	5
	min_samples_leaf	5
	class_weight	balanced
	random_state	42
Gradient Boosting	n_estimators	150
	max_depth	3
	learning_rate	0.05
	min_samples_leaf	5
	subsample	0.8
SVM-RBF	C	1.0
	kernel	rbf
	gamma	scale
	class_weight	balanced
KNN	n_neighbors	7
	weights	distance
1-Layer NN	hidden_layer_sizes	(32,)
	activation	relu
	solver	adam
	max_iter	500
	early_stopping	True
EfficientNet-B0	backbone	Frozen ImageNet pretrained
	head	1280→64→ReLU→Dropout(0.4)→1
	learning_rate	1e-3
	scheduler	CosineAnnealing
	epochs	30
	batch_size	16
	loss	BCEWithLogitsLoss + pos_weight
	augmentation	H-flip, V-flip, Rot(15°), ColorJitter
Gated Fusion	architecture	σ(W·[img‖tab]) ⊙ [img‖tab]
	tab_branch	Linear(n_tab→32)→ReLU
SMOTE	k_neighbors	3
	application	Train folds only (imblearn Pipeline)
CV (ML)	scheme	RepeatedStratifiedKFold
	n_splits × n_repeats	5 × 10 = 50 folds
CV (DL)	scheme	RepeatedStratifiedKFold
	n_splits × n_repeats	5 × 3 = 15 folds
Feature selection	Stage 1	Remove std features
	Stage 2	Remove zero-variance
	Stage 3	Remove correlated (r > 0.95)
Permutation test	n_iterations	100
Bootstrap CI	n_resamples	500
Software	Python	3.11.15
	scikit-learn	1.8.0
	PyTorch	2.x (RunPod)
	imbalanced-learn	0.14.1
	scikit-image	0.26.0
	Seed	42 (all random operations)

Computational environment: Python 3.11.15; NumPy 2·4·6; pandas 3·0·3; scikit-learn 1·8·0; imbalanced-learn 0·14·1; scikit-image 0·26·0; SciPy 1·17·1; matplotlib 3·10·9; seaborn 0·13·2; PyTorch 2·12·0; torchvision 0·27·0; timm 1·0·27. Random seed: 42 for fold generation, model initialisation where applicable, bootstrap resampling, and permutation testing.

Supplementary Table S4. Feature dictionary for all 117 extracted image features (part 1 of 3).

#	Feature Name	Category	Channel(s)	Description	Biological Proxy
1	red_positive_pixels	Intensity	Red	Count of pixels above Otsu threshold	Stain-positive area (total)
2	red_positive_fraction	Intensity	Red	Fraction of image above threshold	Stain coverage
3	red_mean_intensity	Intensity	Red	Mean pixel intensity across channel	Average stain expression
4	red_median_intensity	Intensity	Red	Median pixel intensity	Robust central tendency of expression
5	red_std_intensity	Intensity	Red	Std deviation of pixel intensity	Expression heterogeneity
6	red_integrated_density	Intensity	Red	Sum of all pixel intensities	Total stain signal
7	red_object_count	Morphology	Red	Number of connected components above threshold	Number of stain-positive structures
8	red_mean_area	Morphology	Red	Mean area of connected components (pixels)	Average structure size
9	red_median_area	Morphology	Red	Median area of components	Typical structure size
10	red_std_area	Morphology	Red	Std of component areas	Size heterogeneity
11	red_min_area	Morphology	Red	Smallest component area	Minimum structure size
12	red_max_area	Morphology	Red	Largest component area	Maximum structure size
13	red_total_area	Morphology	Red	Sum of all component areas	Total structural coverage
14	red_area_cv	Morphology	Red	Coefficient of variation of areas (std/mean)	Structural size heterogeneity
15	green_positive_pixels	Intensity	Green	Count of pixels above Otsu threshold	Stain-positive area (total)
16	green_positive_fraction	Intensity	Green	Fraction of image above threshold	Stain coverage
17	green_mean_intensity	Intensity	Green	Mean pixel intensity across channel	Average stain expression
18	green_median_intensity	Intensity	Green	Median pixel intensity	Robust central tendency of expression
19	green_std_intensity	Intensity	Green	Std deviation of pixel intensity	Expression heterogeneity
20	green_integrated_density	Intensity	Green	Sum of all pixel intensities	Total stain signal
21	green_object_count	Morphology	Green	Number of connected components above threshold	Number of stain-positive structures
22	green_mean_area	Morphology	Green	Mean area of connected components (pixels)	Average structure size
23	green_median_area	Morphology	Green	Median area of components	Typical structure size
24	green_std_area	Morphology	Green	Std of component areas	Size heterogeneity
25	green_min_area	Morphology	Green	Smallest component area	Minimum structure size
26	green_max_area	Morphology	Green	Largest component area	Maximum structure size
27	green_total_area	Morphology	Green	Sum of all component areas	Total structural coverage
28	green_area_cv	Morphology	Green	Coefficient of variation of areas (std/mean)	Structural size heterogeneity
29	blue_positive_pixels	Intensity	Blue	Count of pixels above Otsu threshold	Stain-positive area (total)
30	blue_positive_fraction	Intensity	Blue	Fraction of image above threshold	Stain coverage
31	blue_mean_intensity	Intensity	Blue	Mean pixel intensity across channel	Average stain expression
32	blue_median_intensity	Intensity	Blue	Median pixel intensity	Robust central tendency of expression
33	blue_std_intensity	Intensity	Blue	Std deviation of pixel intensity	Expression heterogeneity
34	blue_integrated_density	Intensity	Blue	Sum of all pixel intensities	Total stain signal
35	blue_object_count	Morphology	Blue	Number of connected components above threshold	Number of stain-positive structures
36	blue_mean_area	Morphology	Blue	Mean area of connected components (pixels)	Average structure size
37	blue_median_area	Morphology	Blue	Median area of components	Typical structure size
38	blue_std_area	Morphology	Blue	Std of component areas	Size heterogeneity
39	blue_min_area	Morphology	Blue	Smallest component area	Minimum structure size

Supplementary Table S4. Feature dictionary for all 117 extracted image features (part 2 of 3).

#	Feature Name	Category	Channel(s)	Description	Biological Proxy
40	blue_max_area	Morphology	Blue	Largest component area	Maximum structure size
41	blue_total_area	Morphology	Blue	Sum of all component areas	Total structural coverage
42	blue_area_cv	Morphology	Blue	Coefficient of variation of areas (std/mean)	Structural size heterogeneity
43	red_green_overlap_pixels	Co-localization	R↔G	Pixels positive in both channels	Co-expression area
44	red_green_overlap_fraction	Co-localization	R↔G	Fraction of image with dual positivity	Co-expression coverage
45	red_green_jaccard_index	Co-localization	R↔G	Intersection/Union of positive masks	Spatial overlap similarity
46	red_green_overlap_coeff_1	Co-localization	R↔G	Overlap coefficient (ch1→ch2)	Directional co-occurrence
47	red_green_overlap_coeff_2	Co-localization	R↔G	Overlap coefficient (ch2→ch1)	Directional co-occurrence
48	red_green_pearson_correlation	Co-localization	R↔G	Pearson r of binary masks	Linear spatial correlation
49	red_green_manders_m1	Co-localization	R↔G	Manders' coefficient M1	Fraction ch1 overlapping ch2
50	red_green_manders_m2	Co-localization	R↔G	Manders' coefficient M2	Fraction ch2 overlapping ch1
51	red_green_mean_distance_1to2	Proximity	R↔G	Mean NN distance from ch1→ch2 centroids	Spatial proximity
52	red_green_median_distance_1to2	Proximity	R↔G	Median NN distance ch1→ch2	Typical proximity
53	red_green_std_distance_1to2	Proximity	R↔G	Std of NN distances ch1→ch2	Proximity variability
54	red_green_min_distance_1to2	Proximity	R↔G	Min NN distance ch1→ch2	Closest approach
55	red_green_max_distance_1to2	Proximity	R↔G	Max NN distance ch1→ch2	Farthest separation
56	red_green_nearby_fraction_1to2	Proximity	R↔G	Fraction of ch1 objects within 50px of ch2	Close co-occurrence rate
57	red_green_proximity_index_1to2	Proximity	R↔G	Mean 1/(1+dist) for ch1→ch2	Weighted proximity score
58	red_green_mean_distance_2to1	Proximity	R↔G	Mean NN distance ch2→ch1	Reverse spatial proximity
59	red_green_median_distance_2to1	Proximity	R↔G	Median NN distance ch2→ch1	Reverse typical proximity
60	red_green_std_distance_2to1	Proximity	R↔G	Std NN distances ch2→ch1	Reverse proximity variability
61	red_green_min_distance_2to1	Proximity	R↔G	Min NN distance ch2→ch1	Reverse closest approach
62	red_green_max_distance_2to1	Proximity	R↔G	Max NN distance ch2→ch1	Reverse farthest separation
63	red_green_nearby_fraction_2to1	Proximity	R↔G	Fraction ch2 within 50px of ch1	Reverse co-occurrence
64	red_green_proximity_index_2to1	Proximity	R↔G	Mean 1/(1+dist) ch2→ch1	Reverse proximity score
65	red_blue_overlap_pixels	Co-localization	R↔B	Pixels positive in both channels	Co-expression area
66	red_blue_overlap_fraction	Co-localization	R↔B	Fraction of image with dual positivity	Co-expression coverage
67	red_blue_jaccard_index	Co-localization	R↔B	Intersection/Union of positive masks	Spatial overlap similarity
68	red_blue_overlap_coeff_1	Co-localization	R↔B	Overlap coefficient (ch1→ch2)	Directional co-occurrence
69	red_blue_overlap_coeff_2	Co-localization	R↔B	Overlap coefficient (ch2→ch1)	Directional co-occurrence
70	red_blue_pearson_correlation	Co-localization	R↔B	Pearson r of binary masks	Linear spatial correlation
71	red_blue_manders_m1	Co-localization	R↔B	Manders' coefficient M1	Fraction ch1 overlapping ch2
72	red_blue_manders_m2	Co-localization	R↔B	Manders' coefficient M2	Fraction ch2 overlapping ch1
73	red_blue_mean_distance_1to2	Proximity	R↔B	Mean NN distance from ch1→ch2 centroids	Spatial proximity
74	red_blue_median_distance_1to2	Proximity	R↔B	Median NN distance ch1→ch2	Typical proximity
75	red_blue_std_distance_1to2	Proximity	R↔B	Std of NN distances ch1→ch2	Proximity variability
76	red_blue_min_distance_1to2	Proximity	R↔B	Min NN distance ch1→ch2	Closest approach
77	red_blue_max_distance_1to2	Proximity	R↔B	Max NN distance ch1→ch2	Farthest separation
78	red_blue_nearby_fraction_1to2	Proximity	R↔B	Fraction of ch1 objects within 50px of ch2	Close co-occurrence rate

Supplementary Table S4. Feature dictionary for all 117 extracted image features (part 3 of 3).

#	Feature Name	Category	Channel(s)	Description	Biological Proxy
79	red_blue_proximity_index_1to2	Proximity	R↔B	Mean 1/(1+dist) for ch1→ch2	Weighted proximity score
80	red_blue_mean_distance_2to1	Proximity	R↔B	Mean NN distance ch2→ch1	Reverse spatial proximity
81	red_blue_median_distance_2to1	Proximity	R↔B	Median NN distance ch2→ch1	Reverse typical proximity
82	red_blue_std_distance_2to1	Proximity	R↔B	Std NN distances ch2→ch1	Reverse proximity variability
83	red_blue_min_distance_2to1	Proximity	R↔B	Min NN distance ch2→ch1	Reverse closest approach
84	red_blue_max_distance_2to1	Proximity	R↔B	Max NN distance ch2→ch1	Reverse farthest separation
85	red_blue_nearby_fraction_2to1	Proximity	R↔B	Fraction ch2 within 50px of ch1	Reverse co-occurrence
86	red_blue_proximity_index_2to1	Proximity	R↔B	Mean 1/(1+dist) ch2→ch1	Reverse proximity score
87	green_blue_overlap_pixels	Co-localization	G↔B	Pixels positive in both channels	Co-expression area
88	green_blue_overlap_fraction	Co-localization	G↔B	Fraction of image with dual positivity	Co-expression coverage
89	green_blue_jaccard_index	Co-localization	G↔B	Intersection/Union of positive masks	Spatial overlap similarity
90	green_blue_overlap_coeff_1	Co-localization	G↔B	Overlap coefficient (ch1→ch2)	Directional co-occurrence
91	green_blue_overlap_coeff_2	Co-localization	G↔B	Overlap coefficient (ch2→ch1)	Directional co-occurrence
92	green_blue_pearson_correlation	Co-localization	G↔B	Pearson r of binary masks	Linear spatial correlation
93	green_blue_manders_m1	Co-localization	G↔B	Manders' coefficient M1	Fraction ch1 overlapping ch2
94	green_blue_manders_m2	Co-localization	G↔B	Manders' coefficient M2	Fraction ch2 overlapping ch1
95	green_blue_mean_distance_1to2	Proximity	G↔B	Mean NN distance from ch1→ch2 centroids	Spatial proximity
96	green_blue_median_distance_1to2	Proximity	G↔B	Median NN distance ch1→ch2	Typical proximity
97	green_blue_std_distance_1to2	Proximity	G↔B	Std of NN distances ch1→ch2	Proximity variability
98	green_blue_min_distance_1to2	Proximity	G↔B	Min NN distance ch1→ch2	Closest approach
99	green_blue_max_distance_1to2	Proximity	G↔B	Max NN distance ch1→ch2	Farthest separation
100	green_blue_nearby_fraction_1to2	Proximity	G↔B	Fraction of ch1 objects within 50px of ch2	Close co-occurrence rate
101	green_blue_proximity_index_1to2	Proximity	G↔B	Mean 1/(1+dist) for ch1→ch2	Weighted proximity score
102	green_blue_mean_distance_2to1	Proximity	G↔B	Mean NN distance ch2→ch1	Reverse spatial proximity
103	green_blue_median_distance_2to1	Proximity	G↔B	Median NN distance ch2→ch1	Reverse typical proximity
104	green_blue_std_distance_2to1	Proximity	G↔B	Std NN distances ch2→ch1	Reverse proximity variability
105	green_blue_min_distance_2to1	Proximity	G↔B	Min NN distance ch2→ch1	Reverse closest approach
106	green_blue_max_distance_2to1	Proximity	G↔B	Max NN distance ch2→ch1	Reverse farthest separation
107	green_blue_nearby_fraction_2to1	Proximity	G↔B	Fraction ch2 within 50px of ch1	Reverse co-occurrence
108	green_blue_proximity_index_2to1	Proximity	G↔B	Mean 1/(1+dist) ch2→ch1	Reverse proximity score
109	red_green_fraction_ratio	Ratio	R/G	Red pos fraction / Green pos fraction	Relative stain balance
110	green_red_fraction_ratio	Ratio	G/R	Green / Red fraction ratio	Inverse stain balance
111	red_blue_fraction_ratio	Ratio	R/B	Red / Blue fraction ratio	Stain vs nuclear ratio
112	blue_red_fraction_ratio	Ratio	B/R	Blue / Red fraction ratio	Nuclear vs stain ratio
113	green_blue_fraction_ratio	Ratio	G/B	Green / Blue fraction ratio	Stain vs nuclear ratio
114	blue_green_fraction_ratio	Ratio	B/G	Blue / Green fraction ratio	Nuclear vs stain ratio
115	red_max_distance_from_center	Spatial	Red	Max distance of red object from image center	Spatial spread
116	green_max_distance_from_center	Spatial	Green	Max distance of green object from center	Spatial spread
117	blue_max_distance_from_center	Spatial	Blue	Max distance of blue object from center	Nuclear spread

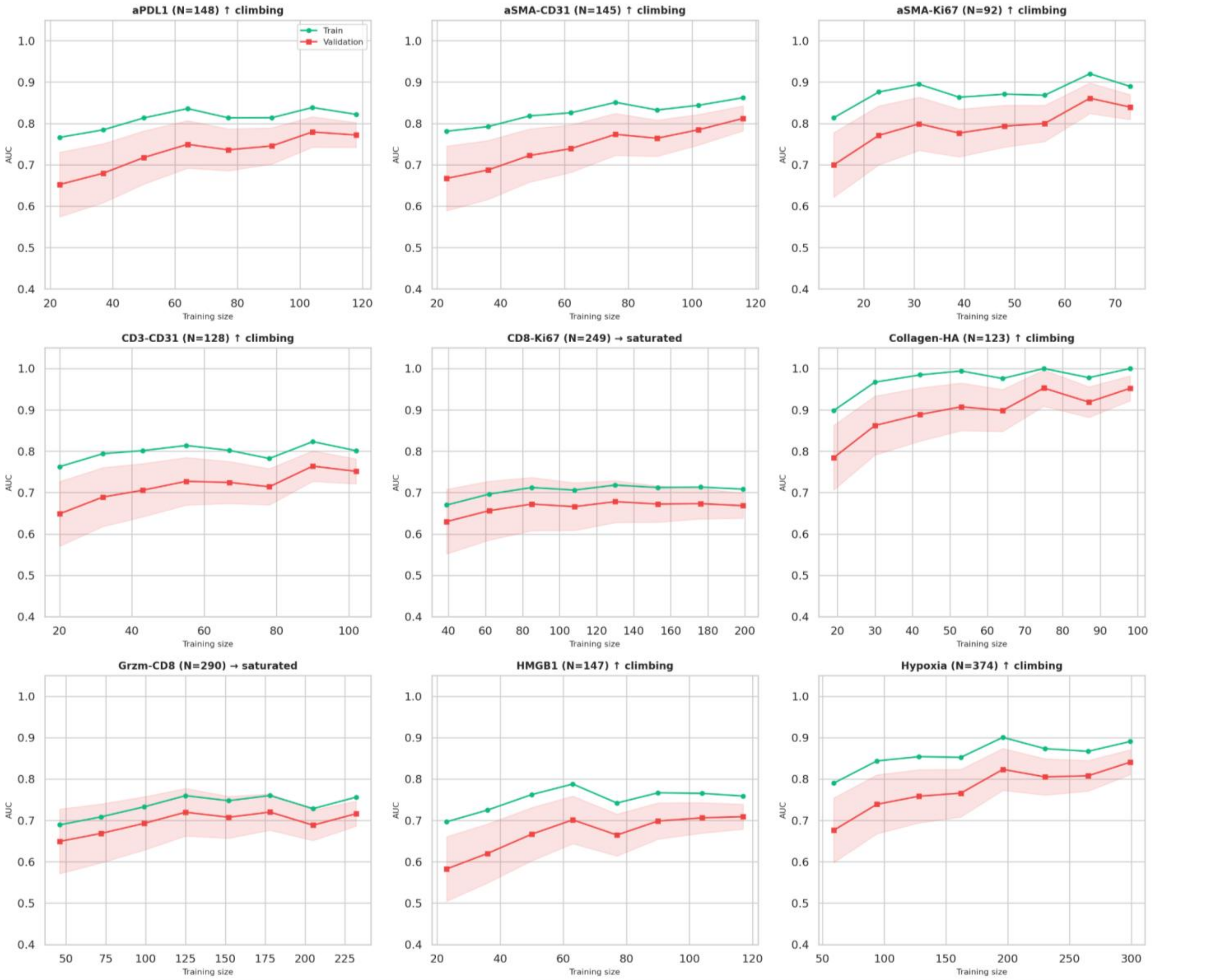

Supplementary Figure S1. Learning curves for all nine staining pairs.

Training and validation AUCs are shown as a function of training-set size for each staining. The figure supports the sample-size interpretation in the main text: seven of nine stainings continued to improve with additional data, whereas Granzyme B-CD8 and CD8-Ki67 appeared closer to saturation.

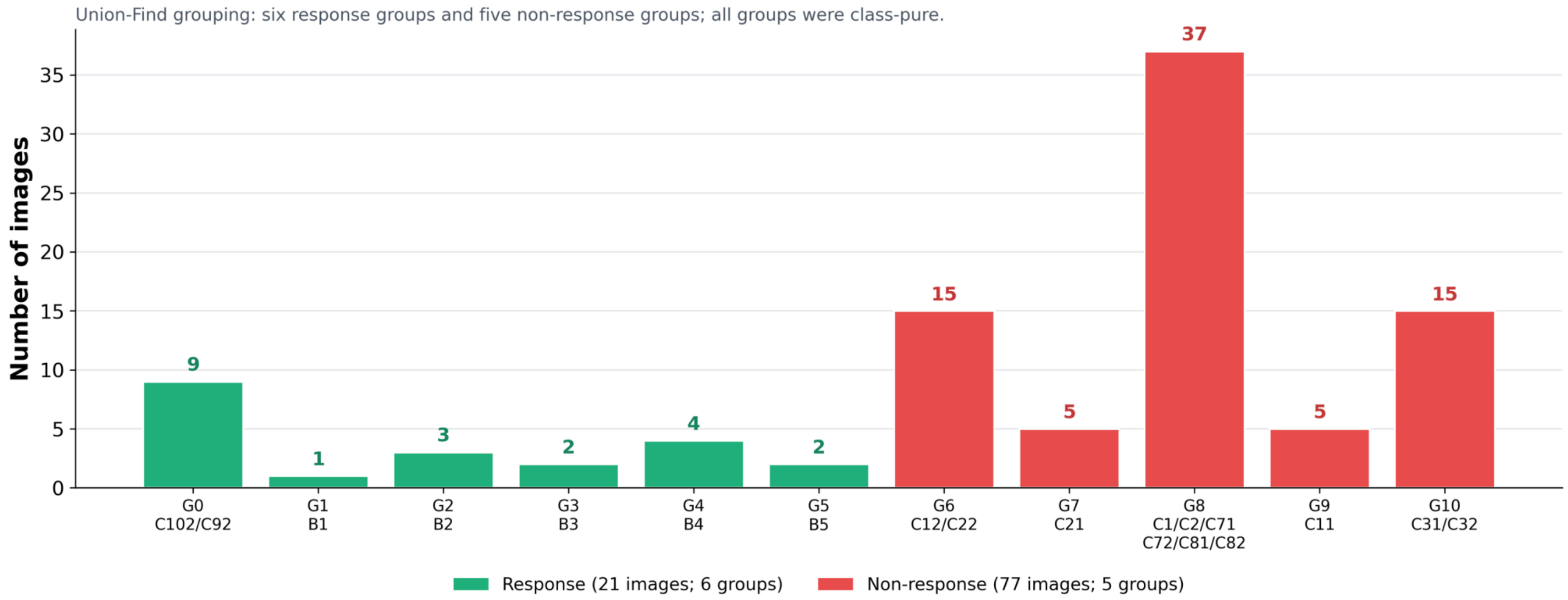

Supplementary Figure S2. Conservative group composition used for GroupKFold leakage-sensitivity analysis.

Bar chart shows the class-pure groups used in the conservative GroupKFold sensitivity analysis. Grouping was used to test whether related image groups could inflate primary cross-validation performance. This figure documents the grouped structure used for the leakage-sensitivity check, not the primary 104-animal class count.
