# Supplementary material for: Computational Pathology Reveals Extracellular-Matrix Imaging Biomarkers of Therapy Response in Preclinical Breast Cancer
Source: Cancers (Basel). 2026 Aug 12;18(16):2603. doi: 10.3390/cancers18162603 (PMC13510428; doi:10.3390/cancers18162603)
Supplement: Supplementary file 1 [file cancers-18-02603-s001.zip › Supplementary Checklist S1. TRIPOD+AI reporting checklist.pdf]

Supplementary Checklist S1 — TRIPOD+AI reporting checklist

Computational Pathology Reveals Extracellular-Matrix Imaging Biomarkers of Therapy Response in Preclinical Breast Cancer

Stelios Lamprou, Styliana Georgiou, Triantafyllos Stylianopoulos, Chrysovalantis Voutouri

TRIPOD+AI (2024) — Transparent Reporting of a multivariable prediction model for Individual Prognosis Or Diagnosis: artificial intelligence extension.

This is a completed draft mapping of the manuscript to the 27 TRIPOD+AI items. Item requirements are summarised in the authors' own words for convenience; before submission, transcribe the Reported/Location entries onto the official TRIPOD+AI checklist (item wording and numbering should be confirmed against the source). Applicability codes: D = development, E = evaluation. This study is a prediction-model DEVELOPMENT with internal cross-validation evaluation in a preclinical (4T1 mouse) setting; there is no external or clinical validation, and several human-oriented items (fairness, patient/public involvement) are Not Applicable.

Official checklist: https://www.tripod-statement.org (Collins GS et al. BMJ 2024;385:e078378)

#	Reporting requirement (summarised)	Reported (Yes/No/NA)	Location in manuscript	Notes
**TITLE AND ABSTRACT**				
1	Title — identify the study as developing and/or evaluating a multivariable prediction model, the target population, and the predicted outcome.	Yes	Title	States prediction of therapy response in preclinical breast cancer.
2	Abstract — structured summary of objectives, data, methods, results (with uncertainty), and conclusions.	Yes	Abstract; Table 1	Background/Methods/Results/Conclusions structure; AUCs in abstract, 95% CIs in Table 1.
**INTRODUCTION**				
3	Background — explain the medical context and rationale, including why the model is needed.	Yes	Background	
4	Objectives — specify the study objectives, including whether the study is development, evaluation, or both.	Yes	Background (final paragraph); Methods	Development + internal evaluation across nine stainings, ECM decomposition, paired fusion.
**METHODS**				
5	Source of data — describe the study design and data source; specify dates.	Yes	Methods — Study design and dataset	Retrospective computational analysis of 4T1 fluorescence IHC datasets.
6	Participants — eligibility/inclusion criteria, setting, and unit of analysis.	Yes	Methods — Sample size, randomisation, masking, and inclusion criteria	Unit = image (benchmark/decomposition) or animal (paired fusion).
7	Data preparation —	Yes	Methods — Image	RGB split, Otsu thresholding, connected

	describe data preprocessing and predictor handling.		preprocessing and Python feature-extraction algorithm	components, 117 features.
8	Outcome — define the outcome being predicted and how/when assessed.	Yes	Methods — Study design	Binary Response vs Non-Response from source experimental datasets.
9	Predictors — define all predictors, including how and when measured.	Yes	Methods — feature extraction; Additional file 4 (Table S4)	117 RGB-channel, intensity, morphometric and cross-channel features.
10	Sample size — explain how study size was determined.	Yes	Methods — Sample size	All eligible images used; no a priori power calculation (stated).
11	Missing data — describe how missing data were handled.	Yes	Methods — inclusion/exclusion; Discussion	Exclusions where matched data unavailable; 276 unmatched Collagen–HA pairs noted.
12	Analytical methods — describe model-building, predictor selection, and internal validation procedures.	Yes	Methods — Multimodal modelling architecture; Feature selection; Validation	Five classical ML + five DL models; 3-stage feature selection; repeated stratified CV.
13	Class imbalance — if addressed, state why and how, and any recalibration.	Yes	Methods — Feature selection and class imbalance	Train-fold-only SMOTE; class-weighted models; stratified folds.
14	Fairness — describe approaches to model fairness and rationale.	N/A — preclinical animal study; no human sensitive attributes. Animal sex unavailable (stated).	Methods	
15	Model output — specify the model output (e.g., probabilities, classification).	Yes	Methods — modelling architecture; Results	Binary classification with probability scores; AUC primary metric.
16	Model performance / evaluation — describe how performance was estimated, including discrimination and calibration.	Yes	Methods — Validation and high-AUC quality control	AUC, balanced accuracy, F1, precision, recall; bootstrap 95% CIs; permutation tests; calibration as exploratory QC.
17	Model updating — describe any recalibration/updating from evaluation.	N/A — no model updating performed.	Methods	
**OPEN SCIENCE**				
18	Open science — report funding, conflicts, protocol/registration, and data/code availability.	Yes (partial)	Declarations; Availability of data and materials	Funding, competing interests, and code (GitHub) reported; study not prospectively registered (not applicable to retrospective computational analysis).
**PATIENT AND PUBLIC INVOLVEMENT**				
19	Patient and public involvement — describe how patients/public were involved.	N/A — preclinical animal study; no patient/public involvement.	—	
**RESULTS**				
20	Participants — describe	Yes	Results — Dataset	Image counts per staining; treatment

	the flow and numbers of participants/units through the study; key characteristics.		and workflow; Figure 1	distribution.
21	Model development — report the final model / feature configurations.	Yes	Results; Table 1; Additional files 1–4	Best models per staining; decomposition and fusion configurations.
22	Model performance — report performance estimates with confidence intervals; key subgroups.	Yes	Results; Table 1	AUCs with 95% CIs, BA, F1, precision, recall for decomposition and fusion analyses.
23	Model evaluation — report results of any internal/external evaluation.	Yes	Results — Validation analyses supported robustness	Permutation tests, GroupKFold leakage analysis, learning curves.
24	Model updating — report results of any model updating.	N/A — none performed.	—	
**DISCUSSION**				
25	Interpretation — overall interpretation of results, including fairness, in context of objectives and prior work.	Yes	Discussion	
26	Limitations — discuss limitations and their effects on bias, uncertainty, and generalisability.	Yes	Discussion	Preclinical only; n=104 (21 responders); internal CV only; no external cohort; features not yet pathologist-validated.
27	Usability and next steps — handling of poor/unavailable input, required user expertise, and future research directions.	Yes	Discussion	External validation and human archival-tissue translation specified as next steps.
