# Supplementary material for: Computational Pathology Reveals Extracellular-Matrix Imaging Biomarkers of Therapy Response in Preclinical Breast Cancer
Source: Cancers (Basel). 2026 Aug 12;18(16):2603. doi: 10.3390/cancers18162603 (PMC13510428; doi:10.3390/cancers18162603)
Supplement: Supplementary file 1 [file cancers-18-02603-s001.zip › Supplementary Checklist S2. CLAIM 2024 reporting checklist.pdf]

Supplementary Checklist S2 — CLAIM 2024 reporting checklist

Computational Pathology Reveals Extracellular-Matrix Imaging Biomarkers of Therapy Response in Preclinical Breast Cancer

Stelios Lamprou, Styliana Georgiou, Triantafyllos Stylianopoulos, Chrysovalantis Voutouri

CLAIM (2024 Update) — Checklist for Artificial Intelligence in Medical Imaging.

This is a completed draft mapping of the manuscript to the CLAIM items. Item requirements are summarised in the authors' own words; before submission, transcribe the Reported/Location entries onto the official CLAIM form. Per CLAIM 2024, each item is marked Yes, No, or Not Applicable (NA), and the term "validation" is avoided in favour of "internal test". This is a preclinical (4T1 mouse) immunofluorescence study; "reference standard" = the experimentally defined Response/Non-Response label; "patients/examinations" map to animals/images.

Official checklist: https://pubs.rsna.org/doi/full/10.1148/ryai.240300 (Tejani AS et al. Radiol Artif Intell 2024;6:e240300; original: Mongan J et al. 2020;2:e200029)

#	Reporting requirement (summarised)	Reported (Yes/No/NA)	Location in manuscript	Notes
**TITLE / ABSTRACT**				
1	Identify the work as a study of AI/ML methodology in the title and/or abstract.	Yes	Title; Abstract	"multimodal machine learning and deep learning" stated.
2	Structured abstract summarising design, data/population, methods, and results.	Yes	Abstract	Background/Methods/Results/Conclusions; image counts and AUCs given.
**INTRODUCTION**				
3	State the scientific/clinical background and the intended role of the AI approach.	Yes	Background	
4	State the study objectives and hypotheses.	Yes	Background (final paragraph)	
**METHODS — Study design**				
5	Prospective or retrospective study; state which.	Yes	Methods — Study design	Retrospective computational analysis.
6	Study goal (e.g., model development, internal test).	Yes	Methods; Background	Development + internal cross-validated evaluation.
**METHODS — Data**				
7	Data sources and acquisition (modality, staining, instrumentation).	Yes	Methods — Study design; Image preprocessing	Fluorescence IHC, nine biomarker stainings.
8	Eligibility/inclusion and exclusion criteria for data.	Yes	Methods — inclusion criteria	Assignable staining, response label, usable RGB channels.
9	Data pre-processing	Yes	Methods —	RGB split, Otsu thresholding, masks,

	steps.		Image preprocessing	connected components.
10	Selection of data subsets, if applicable.	Yes	Methods — Experimental modules	Nine-staining benchmark; 346-image decomposition; 104-animal paired fusion.
11	Definitions of data elements / extracted features.	Yes	Additional file 4 (Table S4)	117-feature dictionary.
12	De-identification / data-handling and ethics.	Yes	Methods — Ethics; Declarations	Animal-derived images; license CY/EXP/PR.L01/2024; no human data.
**METHODS — Reference standard (ground truth)**				
13	Definition of the reference standard used to establish labels.	Yes	Methods — Study design	Binary Response vs Non-Response from source experiments.
14	Rationale for choosing the reference standard.	Yes	Methods; Background	Therapy-response endpoint of source preclinical experiments.
15	Source of reference-standard annotations; qualifications of annotators.	Yes (partial)	Methods	Labels derived from experimental treatment-response outcomes (not subjective image reads).
16	Annotation tools / measurement of inter-rater variability.	N/A — labels are experimentally defined, not manually annotated per image.	—	
**METHODS — Data partitions**				
17	Intended sample size and how determined.	Yes	Methods — Sample size	All eligible images/animals; no a priori calculation.
18	How data were assigned to partitions; level of splitting (image- or animal-level).	Yes	Methods — Validation	Repeated stratified CV; GroupKFold by C-code/animal to test leakage.
19	Level at which partitions are disjoint (to avoid leakage).	Yes	Methods — Validation; Results	Animal-level grouping tested; max AUC reduction 0.052 under GroupKFold.
**METHODS — Model**				
20	Detailed description of model, including architecture(s).	Yes	Methods — Multimodal modelling architecture	RF, GB, SVM, KNN, 1L-NN; EfficientNet, stain-deconvolution, PhiKon; gated fusion.
21	Software/hardware, libraries, and versions.	Yes	Methods — Computational environment; Additional file 3 (Table S3)	Python 3.11.15; scikit-learn, PyTorch 2.12.0, etc.
22	Initialisation of model parameters (e.g., pretrained weights).	Yes	Methods; Additional file 3	ImageNet-pretrained EfficientNet-B0; seeds = 42.
**METHODS — Training**				
23	Training approach, hyperparameters, and optimisation.	Yes	Additional file 3 (Table S3)	Full hyperparameters/fold configuration.
24	Method of selecting the final model / hyperparameters.	Yes	Methods — Feature selection; Validation	3-stage feature selection; CV-based model selection.
25	Handling of class imbalance / data augmentation.	Yes	Methods — Feature selection and class imbalance	Train-fold-only SMOTE; class weights; image augmentation (Table S3).
**METHODS — Evaluation**				

26	Metrics of model performance.	Yes	Methods — Validation; Table 1	AUC, balanced accuracy, F1, precision, recall.
27	Statistical measures of significance and uncertainty (e.g., CIs).	Yes	Methods — Validation; Table 1	Bootstrap 95% CIs; permutation p-values.
28	Robustness / sensitivity analyses.	Yes	Results — Validation analyses; Figure 6	Permutation testing, GroupKFold leakage, feature-stability, learning curves.
29	Methods for explainability / interpretability.	Yes (partial)	Methods — feature extraction; Discussion	Interpretable handcrafted features; channel decomposition for biological interpretation.
30	Validation or testing on external data.	N/A — no external dataset available; internal cross-validation only (stated limitation).	Discussion	
31	Reference to the AI model/code availability.	Yes	Declarations — Availability of data and materials	Code at GitHub repository.
**RESULTS — Data**				
32	Flow of data/cases through the study (numbers at each stage).	Yes	Results — Dataset and workflow; Figure 1	
33	Demographic and clinical characteristics of cases.	Yes (partial)	Methods; Figure 1	Treatment-group distribution; animal sex not available (stated).
**RESULTS — Model performance**				
34	Performance metrics for the model(s) on all partitions.	Yes	Results; Table 1; Additional files 1–2	Per-staining AUCs; decomposition and fusion metrics with CIs.
35	Estimates of diagnostic accuracy / discrimination and their uncertainty.	Yes	Results; Table 1	AUCs with bootstrap 95% CIs.
**DISCUSSION**				
36	Study limitations, including potential bias and generalisability.	Yes	Discussion	Preclinical; small paired n; internal CV only; no external cohort.
37	Implications for practice, including intended use and future work.	Yes	Discussion; Conclusions	External and human-tissue validation specified as next steps.
**OTHER INFORMATION**				
38	Registration number and name of registry.	N/A — retrospective computational analysis; not registered.	—	
39	Where the full study protocol can be accessed.	N/A — no separate protocol; methods fully described in manuscript.	Methods	
40	Sources of funding and role of funders.	Yes	Declarations — Funding	THALIA 2021–2027; funders had no role.
41	Conflicts of interest.	Yes	Declarations — Competing	None declared.

			interests	
42	Ethics approval / animal welfare statement.	Yes	Methods — Ethics; Declarations	Cyprus Veterinary Services license CY/EXP/PR.L01/2024; EU Directive 2010/63/EU; ARRIVE 2.0 [37].
43	Data availability.	Yes	Declarations — Availability of data and materials	Available from corresponding author; subject to institutional requirements.
44	Use of generative AI in the writing process (disclosure).	Yes	Declarations — Use of generative AI...	Declared (no generative AI used).
