# Supplementary material for: Computational Pathology Reveals Extracellular-Matrix Imaging Biomarkers of Therapy Response in Preclinical Breast Cancer
Source: Cancers (Basel). 2026 Aug 12;18(16):2603. doi: 10.3390/cancers18162603 (PMC13510428; doi:10.3390/cancers18162603)
Supplement: Supplementary file 1 [file cancers-18-02603-s001.zip › Supplementary Checklist S3. ARRIVE 2.0 reporting checklist.pdf]

Supplementary Checklist S3 — ARRIVE 2.0 reporting checklist

Computational Pathology Reveals Extracellular-Matrix Imaging Biomarkers of Therapy Response in Preclinical Breast Cancer

Stelios Lamprou, Styliana Georgiou, Triantafyllos Stylianopoulos, Chrysovalantis Voutouri

Checklist status: Completed reporting map based on the currently available manuscript, supplementary material, and Biomarker Research revision. Items that cannot be verified from the available documents are marked as not available or not reported, rather than inferred.

Scope: The current manuscript is a retrospective computational pathology analysis of animal-derived immunofluorescence images from preclinical 4T1 murine breast cancer therapy-response experiments. The checklist therefore maps ARRIVE 2.0 reporting expectations to the animal-derived component and the secondary computational analysis.

Important caution: This document does not invent missing details. Where approval-day information, animal strain/source/age/weight/sex, housing, husbandry, welfare monitoring, endpoint criteria, randomisation/blinding, or acquisition metadata are unavailable from the retrospective computational dataset, the corresponding checklist entries are marked as not available or not reported.

Summary of completion status

Status overview: Partial: 8; Yes/Partial: 2; Partial/No: 1; Yes: 7; No: 1; No/Partial: 1; N/A/No: 1. Items marked Partial/No require author verification from the original animal-study records before final Cancers submission.

ARRIVE set	Item	ARRIVE 2.0 item	Reported?	Manuscript location	Current reporting	Author action / gap
Essential 10	1	Study design	Partial	Methods: Study design and dataset; Figure 1; Table 1	The manuscript states that this was a retrospective computational pathology study of fluorescence immunohistochemistry images from preclinical 4T1 murine breast cancer therapy-response experiments. It lists treatment classes and defines image-level versus animal-level analyses.	Verify full source-experiment design: exact animal numbers per treatment arm, experimental unit, number of independent experiments/batches, and whether controls and treatment groups were balanced at animal level.
Essential 10	2	Sample size	Partial	Methods: Sample size, randomisation, masking, and inclusion criteria; Results; Table 1	All eligible images with assignable staining panel, response label, and usable RGB channels were included. Main dataset: 1,696 images. Collagen-HA decomposition: 346 images. Paired animal-level fusion: 104 animals, including 21 responders and 83 non-responders. No prospective sample-size calculation was	If available, add animal-level sample-size rationale for the original in vivo experiment. Clarify whether the original study had pre-specified group sizes or power calculations.

					performed for the computational analysis.	
Essential 10	3	Inclusion and exclusion criteria	Yes/Partial	Methods: Sample size, randomisation, masking, and inclusion criteria	Images were included if they had an assignable staining panel, response label, and usable RGB fluorescence channels. Animals/images were excluded from paired fusion only when matched hypoxia and Collagen-HA data were unavailable or file identifiers could not be reconciled.	Add whether any original animals/tissues were excluded before imaging and whether exclusion decisions were made blinded to response.
Essential 10	4	Randomisation	Partial	Methods: Sample size, randomisation, masking, and inclusion criteria	The manuscript states that treatment allocation and tissue generation followed the original preclinical experimental protocols. No randomisation method is described in the available manuscript.	Author to verify and report how animals were allocated to control, chemotherapy, immune-checkpoint inhibition, mechanotherapeutic, dual-combination, and triple-combination groups.
Essential 10	5	Blinding	Partial/No	Methods: Sample size, randomisation, masking, and inclusion criteria	The computational image-preprocessing, feature-extraction, and model-training pipelines are described as predefined and automated. Blinding during the original animal experiment, tissue collection, staining, image acquisition, or response-label assignment is not described.	Author to verify whether investigators were blinded during treatment allocation, tumour measurement, tissue processing, image acquisition, image selection, or outcome assessment.
Essential 10	6	Outcome measures	Partial	Methods: Study design and dataset; Results	The predicted outcome is binary therapy Response versus Non-Response, defined in the source experimental datasets. Primary computational metrics are AUC, balanced accuracy, F1 score, precision, recall, bootstrap confidence intervals, and permutation p values.	Author to verify the biological response criterion used in the source experiment: tumour-volume threshold, time point, necrosis/regression criterion, survival endpoint, or other operational definition.
Essential 10	7	Statistical methods	Yes	Methods: Feature selection and class imbalance; Validation and	The manuscript reports repeated stratified cross-validation, train-	Check that all reported analyses correspond exactly to the final scripts and repository.

				high-AUC quality control; Computational environment; Table 1; Supplementary Table S3	fold-only SMOTE, class weighting where supported, bootstrap 95% confidence intervals, permutation testing, GroupKFold leakage sensitivity, calibration and feature-stability analyses where available, and software versions.	
Essential 10	8	Experimental animals	Partial	Introduction/Methods: 4T1 murine breast cancer model; Methods: Sample size section	The model is described as a preclinical 4T1 murine breast cancer therapy-response setting. Animal sex was not available in the computational datasets and was not analysed.	Author to add strain/background, supplier/source, age, weight range, sex if recoverable, immune status, number of animals by group, and relevant health status/acclimatisation details.
Essential 10	9	Experimental procedures	Partial	Methods: Study design and dataset; Ethics; Image preprocessing; Figure legends	The manuscript summarises therapy classes and computational image procedures. It describes RGB-channel parsing, Otsu thresholding, binary masks, connected-component measurements, and fluorescence channels for Collagen I, HA, and DAPI.	Author to add source-protocol details: tumour implantation site/cell number, treatment agents/doses/routes/schedule, tissue collection timing, fixation/sectioning/staining protocol, antibody/reagent information, microscope/objective/camera/detector/filters/acquisition software, magnification/scale bars, and image bit depth/pixel size.
Essential 10	10	Results	Yes/Partial	Results; Table 1; Figures 1-6; Supplementary Tables/Figures	The manuscript reports the number of images/animals analysed and model outcomes across benchmark, decomposition, and paired fusion analyses. It reports missing paired Collagen-HA data as a limitation.	If available, add animal-level attrition from the source experiment and adverse/welfare events. Clarify whether all animals assigned to treatment contributed tissue images.
Recommended Set	11	Abstract	Yes	Abstract	The abstract summarises the preclinical image dataset, computational methods, main AUC findings, and conclusion that ECM histological imaging provides a preclinical therapy-response signal.	For Cancers, retain the structured headings Background/Objectives, Methods, Results, Conclusions.
Recommended Set	12	Background	Yes	Introduction	The manuscript places therapy response in the context of tumour microenvironment, ECM, collagen,	No major ARRIVE-specific action.

					hyaluronic acid, hypoxia, digital pathology, and ML/DL biomarker discovery.	
Recommended Set	13	Objectives	Yes	Introduction; Methods	The objective is to test whether quantitative multi-staining immunohistochemistry analysed with ML/DL can identify tissue features associated with therapy response, with focused ECM decomposition and paired ECM/hypoxia fusion analyses.	No major ARRIVE-specific action.
Recommended Set	14	Ethical statement	Partial	Methods: Animal Ethics and ARRIVE-Related Reporting; Institutional Review Board Statement	The manuscript states that animal-derived studies were conducted in accordance with European Directive 2010/63/EU and Cyprus animal-welfare legislation and were approved by the Cyprus Veterinary Services committee under license CY/EXP/PR.L01/2024.	Author to verify and insert approval date. Confirm exact directive wording and whether the approval body should be described as ethics committee, national competent authority, or equivalent institutional review body.
Recommended Set	15	Housing and husbandry	No	Not reported in available manuscript	Housing and husbandry details are not available in the current manuscript files.	Author to add facility, cage type, group size, bedding/enrichment, light/dark cycle, temperature/humidity if available, diet/water access, acclimatisation, and any pathogen-status information.
Recommended Set	16	Animal care and monitoring	No/Partial	Not reported in available manuscript; Methods limitation statement	The manuscript does not report pain management, welfare monitoring, humane endpoints, adverse events, or euthanasia method.	Author to add welfare monitoring frequency, analgesia/anaesthesia if used, humane endpoint criteria, tumour-burden limits, euthanasia method, and adverse event reporting from source records.
Recommended Set	17	Interpretation/scientific implications	Yes	Discussion; Conclusions	The manuscript interprets ECM morphology as a biologically coherent preclinical therapy-response signal and discusses collagen/HA contributions and hypoxia relationships.	No major ARRIVE-specific action.
Recommended Set	18	Generalisability/translation	Yes	Discussion; Conclusions	The manuscript states that the study is preclinical and hypothesis-generating, with translation requiring external validation and human archival breast cancer	No major ARRIVE-specific action.

					tissue testing.	
Recommended Set	19	Protocol registration	N/A/No	Not reported	No prospective animal-study protocol registration is reported. This was a retrospective computational secondary analysis.	If the original in vivo animal study was preregistered or had a registered protocol, add registry/name/identifier. If not, state not registered if the journal asks.
Recommended Set	20	Data access	Partial	Data Availability Statement; Code availability; Supplementary Materials	The manuscript states that datasets are available from the corresponding author on reasonable request, subject to institutional data-sharing requirements, and gives a GitHub repository for code.	Author to confirm raw/unprocessed microscopy-image availability, processed feature-table availability, repository status, DOI/archive if possible, and whether institutional restrictions apply.
Recommended Set	21	Declaration of interests	Yes	Conflicts of Interest; Funding; Acknowledgments	The manuscript includes funding information, funder-role statement, and conflict-of-interest statement.	Ensure that industry/commercial relationships involving AnaBioSi-Data Ltd. and any IP/commercialisation context are disclosed if relevant.
